# Evaluating Performance of Microwave Image Reconstruction Algorithms: Extracting Tissue Types with Segmentation Using Machine Learning

**DOI:** 10.3390/jimaging7010005

**Published:** 2021-01-07

**Authors:** Douglas Kurrant, Muhammad Omer, Nasim Abdollahi, Pedram Mojabi, Elise Fear, Joe LoVetri

**Affiliations:** 1 Department of Electrical and Computer Engineering, Schulich School of Engineering, University of Calgary, Calgary, AB T2N 1N4, Canada; muhammad.omer@circlecvi.com (M.O.); fear@ucalgary.ca (E.F.); 2 Department of Electrical and Computer Engineering, Faculty of Engineering, University of Manitoba, Winnipeg, MB R3T 5V6, Canada; abdollan@myumanitoba.ca (N.A.); pedram.mojabi@gmail.com (P.M.); joe.lovetri@umanitoba.ca (J.L.)

**Keywords:** breast imaging, microwave imaging, image reconstruction, segmentation, unsupervised machine learning, *k*-means clustering, Kolmogorov-Smirnov hypothesis test, statistical inference, performance metrics, contrast source inversion

## Abstract

Evaluating the quality of reconstructed images requires consistent approaches to extracting information and applying metrics. Partitioning medical images into tissue types permits the quantitative assessment of regions that contain a specific tissue. The assessment facilitates the evaluation of an imaging algorithm in terms of its ability to reconstruct the properties of various tissue types and identify anomalies. Microwave tomography is an imaging modality that is model-based and reconstructs an approximation of the actual internal spatial distribution of the dielectric properties of a breast over a reconstruction model consisting of discrete elements. The breast tissue types are characterized by their dielectric properties, so the complex permittivity profile that is reconstructed may be used to distinguish different tissue types. This manuscript presents a robust and flexible medical image segmentation technique to partition microwave breast images into tissue types in order to facilitate the evaluation of image quality. The approach combines an unsupervised machine learning method with statistical techniques. The key advantage for using the algorithm over other approaches, such as a threshold-based segmentation method, is that it supports this quantitative analysis without prior assumptions such as knowledge of the expected dielectric property values that characterize each tissue type. Moreover, it can be used for scenarios where there is a scarcity of data available for supervised learning. Microwave images are formed by solving an inverse scattering problem that is severely ill-posed, which has a significant impact on image quality. A number of strategies have been developed to alleviate the ill-posedness of the inverse scattering problem. The degree of success of each strategy varies, leading to reconstructions that have a wide range of image quality. A requirement for the segmentation technique is the ability to partition tissue types over a range of image qualities, which is demonstrated in the first part of the paper. The segmentation of images into regions of interest corresponding to various tissue types leads to the decomposition of the breast interior into disjoint tissue masks. An array of region and distance-based metrics are applied to compare masks extracted from reconstructed images and ground truth models. The quantitative results reveal the accuracy with which the geometric and dielectric properties are reconstructed. The incorporation of the segmentation that results in a framework that effectively furnishes the quantitative assessment of regions that contain a specific tissue is also demonstrated. The algorithm is applied to reconstructed microwave images derived from breasts with various densities and tissue distributions to demonstrate the flexibility of the algorithm and that it is not data-specific. The potential for using the algorithm to assist in diagnosis is exhibited with a tumor tracking example. This example also establishes the usefulness of the approach in evaluating the performance of the reconstruction algorithm in terms of its sensitivity and specificity to malignant tissue and its ability to accurately reconstruct malignant tissue.

## 1. Introduction

Medical imaging with microwave tomography is investigated for breast health monitoring to complement X-ray mammography. For a typical imaging scenario, a multi-illumination approach is implemented by encircling the breast with antennas. The breast is successively illuminated by incident electromagnetic fields from different directions and the resulting scattered and transmitted fields are received by antennas positioned on the breast’s periphery and recorded by the measurement system. Microwave tomography is a model-based imaging modality that extracts internal tissue information from these data to reconstruct an approximation of the actual spatial distribution of the dielectric properties over a reconstruction model consisting of discrete elements. With microwave tomography, bulk tissue characterization is the goal rather than more detailed depiction at the cellular level.

**Figure 1:**
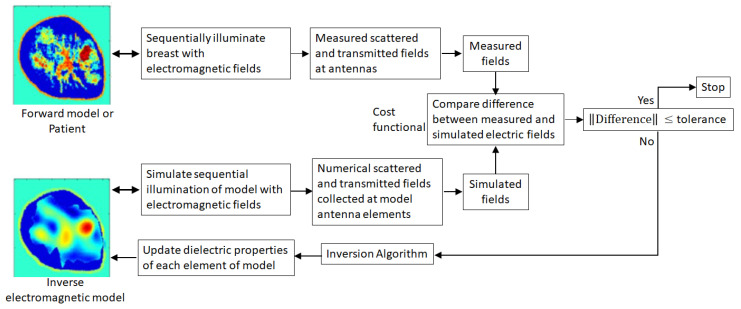
Microwave breast imaging procedure. A breast (represented by a forward model for a numerical study or measurements of a patient) is successively illuminated by incident fields from different directions. Microwave tomography is a model-based modality that extracts internal tissue information from the resulting scattered and transmitted fields to iteratively reconstruct an approximation of actual spatial distribution of dielectric properties of tissues in the breast interior. Different tissue types are distinguished from each other by their characteristic dielectric properties.

The dielectric properties of the breast tissues are represented by a complex permittivity where the real and imaginary components infer the ability of the tissue to store and absorb microwave energy, respectively [1]. The breast tissue types corresponding to skin, adipose (or fatty), transition, fibroglandular, and malignant tissues are characterized by their dielectric properties, which is supported by a number of large-scale studies [2,3,4,5,6,7]. Therefore, the complex permittivity profile that is reconstructed to form an image may be used to distinguish different tissue types. Estimating values of the dielectric properties of tissues over the model in order to reconstruct an image of the interior of the breast is achieved by solving an inverse scattering problem. The inverse problem is non-linear, so the model values are estimated iteratively using a process summarized in Figure 1.

Evaluating approaches to medical image reconstruction requires application of effective metrics to compare different techniques and assess results. Microwave image reconstruction with tomography typically produces lower resolution images than clinical imaging methods such as X-ray. For simulations of known models or experiments with simple phantoms, direct comparisons between microwave images and known values (i.e., comparing the dielectric properties of the forward model with the inverse model shown in Figure 1) have been reported [8,9,10]. This includes examination of cross-sections through models, the average of the error at all points in the image, or the similarity between the spatial distribution of the known dielectric properties of the forward model and the dielectric properties estimated at each of the reconstruction model elements of the inverse model.

For more complex models or clinical cases, evaluation of images is often performed through visual comparison or interpretation based on the clinical history of the patient [11,12]. Quantitative assessment of microwave images is more consistent and precise than a qualitative approach. For evaluating variants of algorithms, assessing the accuracy of reconstructing different tissue types provides detailed insight into the algorithm’s performance.

A more precise and consistent approach to image analysis may be carried out by automatically detecting regions of interest corresponding to various tissue types or anomalies. Accordingly, this necessitates methods capable of distinguishing between different tissue types and anomalies to assist with image interpretation and tumor localization. Moreover, segmenting reconstructed images into tissue types leads to the decomposition of the breast interior into disjoint tissue masks. Metrics are applied to compare masks extracted from reconstructed images and ground truth models. The quantitative results may be used to reveal the accuracy with which the geometric and dielectric properties are reconstructed in order to provide important insights into the performance of the reconstruction algorithm.

Segmenting images formed with microwave tomography can be challenging, as the images may have spurious artefacts and the interfaces that delineate tissue types may be blurred or incorrectly located. In addition, there may be a great deal of inhomogeneity amongst the same tissue type that is reconstructed, inconsistent mapping between estimated dielectric property values of the reconstructed model elements and the range of dielectric properties that characterize a tissue type, and differences in electrical properties reconstructed with variants of an algorithm [8,13,14,15,16].

The segmentation of images into different types of tissues is commonly accomplished using a simple thresholding technique (e.g., [16,17]), whereby reconstructed model elements are classified using ranges of values. However, this strategy assumes that there is a direct mapping between the dielectric property value of a model element estimated by the algorithm and the true dielectric property value of a corresponding tissue type. In practice, this is not necessarily the case, as the accuracy with which the dielectric profile is estimated is impacted by numerous factors, including the number of iterations, the distribution and density of the tissue properties, and measurement parameters (e.g., frequency, number of sensors). Another challenge related to the use of a threshold is that adjustment of the threshold value may significantly impact the specificity and sensitivity to various tissue types. Here, sensitivity and specificity do not refer to the performance of the microwave imaging algorithm in the context of a population of patients, but rather in terms of ability to accurately reconstruct malignant tissues. This problem is apparent when segmenting malignant from healthy tissues and is described in more detail in [17]. Collectively, these problems lead to inconsistent results that contribute to unreliable quantitative assessment of reconstructed images.

An unsupervised machine learning approach such as simulated annealing [18], or *k-*means clustering may be used for image segmentation. However, it is a challenge to determine the optimal number of clusters for the segmentation. Strategies for achieving this task include the elbow method [19], the average silhouette method [20], and the gap statistic method [21]. The elbow technique is a heuristic approach, and an “elbow” could not be unambiguously identified. For many of the images, a great deal of heterogeneity of the reconstructed dielectric properties was observed. This was particularly apparent for images formed from data generated from the heterogeneously dense, scattered density, and extremely dense breasts. The silhouette and gap methods lead to a large range of values that consistently implied a very large number of clusters to partition each image. Consequently, it was not possible to reliably implement any of these methods.

In order to address this problem, this paper presents an iterative approach that does not require the number of clusters to be pre-selected. This is accomplished with an unsupervised machine learning technique that is reinforced with hypothesis testing and statistical inference.

The proposed segmentation algorithm presented in Section 2 is comprised of an iterative clustering method that delineates the interior of the breast into regions dominated by fatty, transition, fibroglandular, and malignant tissues. This segmentation leads to the decomposition of the interior into disjoint tissue masks that are incorporated into a framework whereby both region and distance-based metrics assess image quality [22]. The metrics presented in Section 2 may be used for evaluating variants of reconstruction algorithms, as assessing the accuracy of reconstructing different tissue types provides detailed insight into the algorithm’s performance. Specifically, the segmentation algorithm is applied to forward models and the corresponding microwave images reconstructed with the finite element method contrast source inversion (FEM-CSI) approach. Applying the metrics to the segmentation results allows for comparison between the reconstruction and the original model. Section 3 presents, analyzes, and discusses these results. Finally, conclusions and future explorations are presented in Section 4.

## 2. Methodology

### 2.1. Microwave Images

A high-level depiction of a typical microwave imaging algorithm is illustrated in Figure 1. Although not shown, the breast is encircled with antennas to permit the breast to be illuminated from a variety of locations and directions. Imaging is carried out in two steps. In the first step, the breast is illuminated successively with incident electromagnetic fields from each of the antennas. Hence, the breast is interrogated from multiple directions, and the resulting scattered and transmitted fields are received by antennas located on the breast’s periphery and recorded by the measurement system (see [10,12,15,23,24,25,26,27,28,29], for examples). For a numerical experiment, an electromagnetic forward model comprised of tissues with dielectric properties reported from large-scale studies [2,3,4,5,6,7] is constructed with the techniques described in [30,31]. The model is sequentially illuminated with numerical incident fields, and the calculated scattered and transmitted fields received by the numerical antenna are stored.

Once the experimental data are collected, the reconstruction step using the inversion algorithm is carried out. This second step starts with a trial guess of the distribution. The electromagnetic model of the breast is initialized with this guess. An array of numerical antennas within a simulated measurement chamber that approximates the actual experimental system surrounds the breast and sequentially illuminates the breast with numerical incident fields. The resulting calculated scattered and transmitted fields received at the numerical antennas are recorded. A cost functional measures the discrepancy between the measured and calculated fields, and an inverse solver computes the optimal change in the parameter profile of the electromagnetic model necessary to reduce the discrepancy between these data. The trial solution is updated with these changes, and the forward solver recalculates the electric fields. The process continues in this iterative manner—updating and refining the reconstructed profile—until the calculated and measured fields match which, in turn, implies that the reconstructed profile matches the actual profile.

Various inverse solvers used have been proposed, including the finite element method contrast source inversion (FEM-CSI) [16,32,33], Gauss–Newton method, and conjugate gradient least squares (CGLS) algorithm [34], conjugate gradient method [13], a full-wave inversion method based on wavelet transform [35], wavelet expansion [36], the Distorted Born iterative method [8,37], and an inversion method based on an inexact Newton-type algorithm [38]. A significant challenge encountered when implementing these inverse solvers is that the inverse scattering problem, along with being non-linear, is severely ill-posed. This occurs due to the very large number of elements used by the reconstruction model to capture fine spatial features of the breast. Meanwhile, there are a very limited number of independent measurement data. Hence, the number of reconstruction elements (i.e., the dimension of the solution space) far exceeds the number of independent data resulting in non-unique solutions. An ill-posed inverse problem manifests as small perturbations of the measurement data leading to large errors in the reconstructions, and the convergence to false solutions that fit the data but differ significantly from the actual solution.

To alleviate the ill-posedness of the inverse problem, reconstruction techniques typically incorporate prior information into the objective function by using some form of regularization. The form of regularization used in this paper to improve image quality is to assimilate patient-specific information related to the electrical properties and anatomical structures of the breast into the inhomogeneous background [16,17,33]. The integration of the patient-specific information into the inhomogeneous background reduces the discrepancy between the background complex permittivity and the complex permittivity of the actual profile. In this manner, the patient-specific information serves to encourage convergence to the actual solution and generally reduces the degree of ill-posedness of the inverse scattering problem to improve the stability of the solution [16,39]. Moreover, the size of the solution space is reduced by constraining the size of the imaging domain (or reconstruction model) with knowledge of an estimation of the skin surface location.

Numerical experiments using realistic breast models based on MRI scans [30,40] are tested in this paper, which is depicted in Figure 1 as an electromagnetic forward model. The dielectric properties of the breast are reconstructed from scattered electromagnetic fields by solving an inverse scattering problem using a variant of the finite element method contrast source inversion (FEM-CSI) algorithm [16,33]. Structural information about the breast is introduced into the FEM-CSI algorithm as an inhomogeneous background *ϵ_b_*(**r**). Results are formed by iteratively reconstructing the contrast profile given by,

(1) $$\chi \left(r\right)=\{\begin{array}{cc} \frac{\epsilon \left(r\right)-\epsilon_{b}\left(r\right)}{\epsilon_{b}\left(r\right)}, & r\in D\\ 0, & r\notin D \end{array},$$

where *χ*(**r**) is the contrast profile, *ϵ_b_*(**r**) is the inhomogeneous background profile, *ϵ*(**r**) is the complex permittivity profile, **r** is a position vector, and 𝒟 is the imaging domain bound by boundary ∂𝒟.

**Figure 2:**
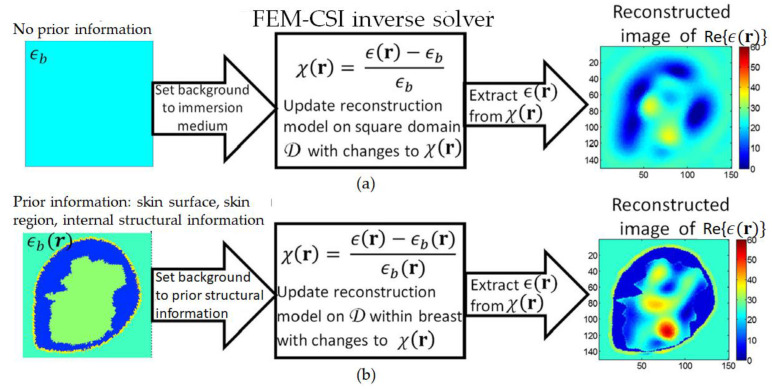
(**a**) With no prior information, background set to immersion medium dielectric properties, and contrast profile reconstructed over square imaging domain. (**b**) Prior information includes skin surface, skin region, and internal structural information. By identifying the breast surface, the imaging domain is constrained to the breast interior.

The use of the background profile to incorporate prior structural information is illustrated in Figure 2. Figure 2a depicts the scenario where there is no structural prior information available, only knowledge of the dielectric properties of the immersion medium. This is equivalent to using the immersion background as the trial solution. This lack of prior information impacts the quality of the resulting microwave image, as the inversion algorithm converges to a solution having low image quality. On the other hand, Figure 2b portrays the case where prior structural information is available. The improvement in the quality of regularization leads to the convergence to a solution associated with a higher image quality relative to the case represented in Figure 2a.

For this study, the FEM-CSI algorithm is terminated once the reconstructed image has stabilized. For example, this may be sensed using the methodology described in [16] or by adapting the technique presented in [41]. The complex permittivity profile is recovered from the contrast profile by using the background permittivity with the relation,

(2) $$\epsilon \left(r\right)=\epsilon_{b}\left(r\right)\left(\chi \left(r\right)+1\right).$$

Using Equations (1) and (2), a list of images of the reconstructed profile is created: the real component of the complex permittivity (Re{*ϵ*(**r**)}), the imaginary component of the complex permittivity (Im{*ϵ*(**r**)}), and the magnitude of the complex permittivity (|*ϵ*(**r**)|), which is a non-linear mapping of the real and imaginary components. Each image is segmented separately using the algorithm described in the following sections.

### 2.2. Segmenting Interior into Healthy and Malignant Breast Tissue Types

The first aim of the segmentation algorithm is to recover the region containing model elements corresponding to malignant tissue (or tissues of interest). The current image of interest is denoted as ℐ. First, the region of interest (breast interior) is defined. The boundary ∂D of the imaging domain 𝒟 given in Equation (1), where D⊂ℐ, is identified. The boundary of a region of interest ∂ℛ is constructed by uniformly contracting ∂𝒟 inward toward the center of 𝒟 by some amount (e.g., 3.5 mm) using the morphological contraction method described in [42,43]. This allows artefacts on the periphery of the imaging domain to be excluded from analysis. The mask of the region ℛ bound by ∂ℛ is constructed such that,

(3) $$mask_{ℛ}=\{\begin{array}{c} 1,\;r\in ℛ\\ 0,\;otherwise \end{array}.$$

Hence, the region of interest ℛ⊂D is extracted from ℐ, with

(4) $$ℛ=mask_{ℛ}⊙ℐ.$$

**Figure 3:**
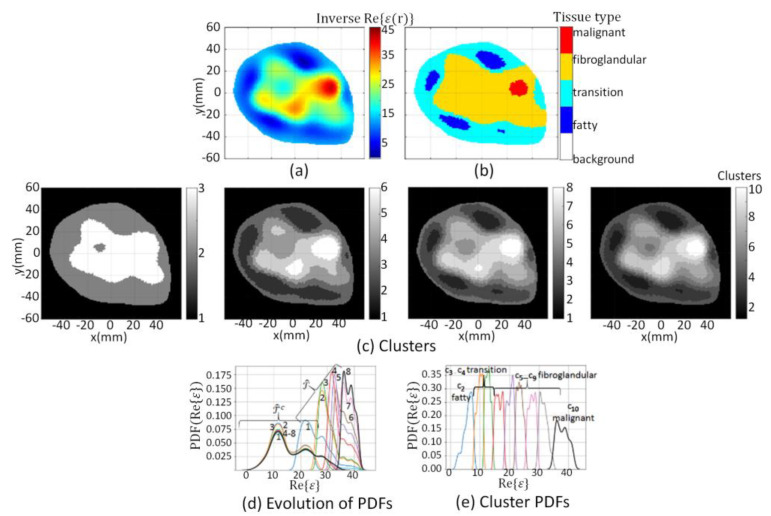
(**a**) Reconstructed component extracted from ℐ=Re{ϵ(r)}; (**c**) Evolution of clusters at *k* = 3, 6, 8, and 10 when segmentation algorithm applied to ℬ; (**d**) Evolution of Probability Density Function (PDF) over data within ${\hat{T}}^{c}$ and $\hat{T}$ where numbers indicate iteration; (**e**) PDF over data within clusters $c_{2}$ (blue line) to $c_{10}$ (black line). Cluster $c_{2}$ corresponds to fatty tissue, $c_{3}-c_{4}$ transition tissue, $c_{5}-c_{9}$ fibroglandular tissues, and $c_{10}$ corresponds to malignant tissue, which are mapped to segmentation masks leading to tissue type image (**b**).

All model elements outside ℛ are assigned a value of −100. An example of ℛ recovered from a reconstructed image that used this contraction method is shown in Figure 3a. Note that the immersion medium and skin are considered as background; only the region of the breast that is interior to the skin is partitioned into tissue types.

Next, the *k-*means clustering technique [44] is iteratively applied to ℬ, where$\;ℬ=ℛ\cup^{\;}{ℛ}^{c}$. The number of clusters *k* is initialized to three, and the *k*-means++ algorithm presented in [45] is used to initialize *k* model elements as cluster centroids. This leads to the delineation of ℛ into clusters *k* = 2 and 3, while the background is outside of ℛ and is assigned cluster *k* = 1. This initial segmentation of ℬ is shown in the left-most panel of Figure 3c. Note that the color bar for Figure 3c corresponds to the number of clusters used for the segmentation. An initial coarse estimate of the tumor region $\hat{T}$ is identified with those model elements assigned the highest value, so $\hat{T}=c_{3}$. Since cluster $c_{2}$ is within ℛ but outside of$\;\hat{T}$, ${\hat{T}}^{c}=c_{2}$. Lastly, the background is outside of ℛ and is always assigned to cluster *k* = 1, which means that$\;{ℛ}^{c}=c_{1}$.

**Figure 4:**
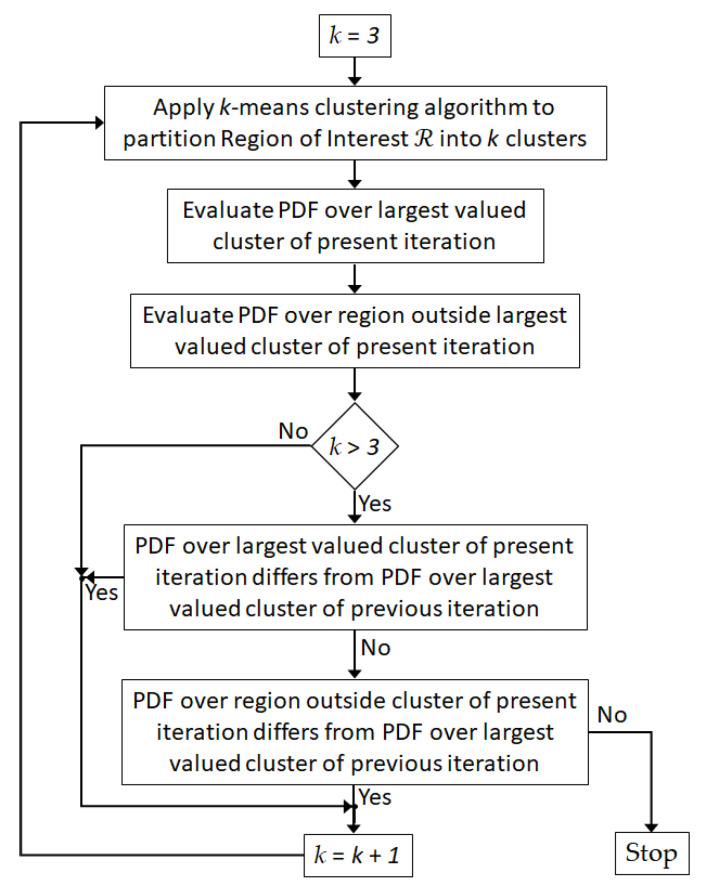
Flow diagram of segmentation algorithm used to refine partitioning of breast interior.

An iterative approach is used to refine $\hat{T}\;$and$\;{\hat{T}}^{c}$, so that with each iteration, the number of clusters *k* used in the *k-*means clustering algorithm is incremented by one. The iterative clustering technique is summarized by Figure 4. After each iteration, $\hat{T}$ and ${\hat{T}}^{c}$ are updated: $\hat{T}$ corresponds to the cluster with the highest-valued integer (i.e., $\hat{T}=c_{\max\left(k\right)}$), while the union of clusters $c_{k}$ with *k* = {2, 3, …, *max*(*k*) − 1} form ${\hat{T}}^{c}$. At each iteration *k*, the mask ${\hat{T}}^{c}$ is applied to the reconstructed image to extract model elements$\;v_{ck}$:

(5) $$\begin{array}{cc} v_{ck}= & \left(\overset{\max\left(k\right)-1}{\underset{k=2}{\cup }}c_{k}\right)⊙ℐ\\ & ={\hat{T}}^{c}⊙ℐ. \end{array}$$

The iterative progression of the segmentation process is demonstrated in Figure 3c whereby clustering results are shown from left-to-right for *k* = 3, 6, 8, and 10.

The empirical distribution function (E(·)) is applied to $v_{ck}$. When *k* > 3, a Kolmogorov–Smirnov (KS) two sample nonparametric hypothesis test evaluates the difference between the cumulative density functions (CDF) of the distributions of the two sample data [46,47]. The test is applied to E($v_{ck}$) and E($v_{ck-1}$) where $v_{ck-1}$ are model elements extracted over ${\hat{T}}^{c}\;$from the previous iteration. The test evaluates the null hypothesis (H_O1_) that $v_{ck}$ and $v_{ck-1}\;$come from the same distribution. Note that the test does not specify the form of the common distribution (e.g., normal distribution). Likewise, the mask $\hat{T}$ is applied to the reconstructed image to extract model elements $v_{tk}$, where $v_{tk}=\hat{T}⊙ℐ.$ In this case, the KS two-sample test is performed on E($v_{tk}$) and E($v_{tk-1}$) to test the null hypothesis (H_O2_) that $v_{tk}$ and $v_{tk-1}$ come from the same distribution. A significance level of 1% is used for both tests.

If either H_O1_ or H_O2_ is rejected, then the number of clusters is incremented by one, and the partitioning procedure is repeated until neither H_O1_ nor H_O2_ is rejected. When neither hypothesis is rejected, this step is terminated. The union of clusters $c_{2}-c_{max\left\{k\right\}-1}$ form$\;{\hat{T}}^{c}$, while $c_{max\left\{k\right\}}$ forms $\hat{T}.$ The probability density function (PDF) over data within ${\hat{T}}^{c}$ and $\hat{T}$ after each iteration is demonstrated in Figure 3d. Convergence of the PDFs is apparent after eight iterations (i.e., *k* = 10, since the segmentation process starts with *k* = 3), which leads to 10 disjoint clusters. Individual PDFs over data within each cluster $c_{2}-c_{8}$ are shown in Figure 3e.

In terms of complexity, finding the global optimum of the *k-*means objective function is a Non-Deterministic Polynomial acceptable (or NP-hard) problem [48,49]. To avoid solving the NP-hard problem, as already indicated, the Lloyd’s clustering algorithm [44] is used but offers a local search heuristic for *k-*means. Given enough time, the algorithm always converges after *i* iterations, but it may be a local minimum. Hence, the clustering algorithm is run multiple times *d* with different initializations of the centroids for each *k*. Then, the result that leads to the smallest objective function value is selected. The *k*-means++ initialization scheme is implemented to reduce the dependence of the initialization of the centroids on the convergence behavior [45].

The running time to implement the proposed segmentation technique is *O(IkidN)*; where *I* is the *n* by *m* image being processed, *k* is the number of clusters, *i* is the number of iterations of the *k-*means clustering algorithm needed until convergence, *d* is the number of times the clustering algorithm is repeated (i.e., find the result leading to the smallest valued objective function after running the algorithm *d* times), and *N* is the number of iterations of the segmentation algorithm required to partition the breast interior. This formulation is derived from [50] and [51], and it includes *N*, which is necessary to implement the segmentation algorithm. The process is repeated for the real component, imaginary component, and the magnitude of the complex permittivity.

For images with large dimensions (i.e., large *n* by *m*), parallel schemes may be implemented in python with the Scikit learn machine learning library (class sklearn.cluster.KMean) that use OpenMp to process small blocks of data in parallel, or Matlab in which the number of times *d* that the *k*-means algorithm is repeated is run in parallel. For the images presented, the data has an underlying clustering structure, and it was observed that the number of iterations *i* of the clustering algorithm until convergence was often small.

### 2.3. Mapping Clusters to Segmentation Masks and Tissue Types

So far, tissues corresponding to model elements with the highest values within the breast are identified by $\hat{T}=c_{\max\left\{k\right\}}$. Cluster $c_{1}\;$identifies the background ${ℛ}^{c}$. The remaining *k* − 2 clusters are mapped to segmentation masks as follows. Cluster $c_{2}$ bounds tissue having the lowest dielectric properties and corresponds to the lowest permittivity values within the breast interior. Consequently, it is reasonable to map $c_{2}\;$to the segmentation mask corresponding to fatty tissue. Next, clusters $c_{3}\;$and $c_{4}\;$contain permittivity values that are higher than fatty tissue. The breast interior includes permittivity values that exceed the maximum value of adipose tissue but are lower than the minimum of the fibroglandular tissue range [3]. Therefore, $c_{3}$ and $c_{4}$ are mapped to a transition segmentation mask. When *max*{*k*} > 4, the union of $c_{5}$ to $c_{\max\left\{k\right\}-1}$ corresponds to segmentation mask $\hat{G}$ associated with fibroglandular tissues. This is defined as:

(6) $$\hat{G}=\left(\cup_{k=5}^{\max\left(k\right)-1}c_{k}\right).$$

The final segmentation is comprised of masks formed by mapping clusters *k* = 1, 2, … *max*{*k*} to tissue types with the function

(7) $$s\left(k\right)=\{\begin{array}{c} background,\;k=1,\\ fatty,\;k=2,\\ \begin{array}{c} transition\;k=3,\;4,\\ \begin{array}{c} fibroglandular,\;4<k<\max\left\{k\right\},\\ malignant,\;k=\max\left\{k\right\}. \end{array} \end{array} \end{array}$$

For the unusual case that there is only one iteration of the segmentation algorithm, clusters $c_{k}$, *k* = 2, 3, 4, are used to identify the fatty, fibroglandular, and malignant tissues, respectively.

The segmentation algorithm is applied to both the forward model and reconstructed images. The resulting segmentation masks are labeled as $ref_{mask}$ and $rec_{mask}$, respectively. To extract the corresponding property values, the reference mask is applied to the forward model. These segmented property values are referred to as the reference tissue, $ref_{tissue}$. Likewise, the reconstructed masks are applied to the reconstructed images. These segmented property values are referred to as the reconstructed tissue, $rec_{tissue}$, of the region. An example of the mapping of the clusters to tissue types is shown in Figure 3b. For this example, the ten clusters shown in the far-right panel of Figure 3c are mapped to segmentation masks and associated tissue types using Equation (7), resulting in the segmented image shown in Figure 3b. Videos demonstrating the iterative refinement of the clusters and segmentation process are provided in the supplemental materials [52].

### 2.4. Quality Assessment

To measure the image reconstruction performance quantitatively, five region-based metrics are applied to assess the overlap between $ref_{mask}$ and $rec_{mask}$. A distance-based metric is also used to evaluate shape fidelity.

First, the accuracy of the geometry of a tissue group is evaluated with [16]

(8) $$\mathrm{Fidelity}\left(ref_{mask},rec_{mask}\right)=\frac{ref_{mask}{}^{T}rec_{mask}\;}{\|ref_{mask}{\|}_{2}\|rec_{mask}{\|}_{2}},\;$$

where the two 2D masks to be compared are first vectorized. The Fidelity value varies from 0 (no similarity) to 1 (perfect similarity). Distortion of the structure and the presence of artefacts decrease the value of this metric. This metric is useful for evaluating the reconstruction of the fibroglandular region.

The next metric evaluates the accuracy with which both the geometric and dielectric properties of the underlying structures are reconstructed. This is measured using the normalized cross-correlation function (xCorrDiel) given by Equation (8), except that $ref_{mask}$ and $rec_{mask}$ are replaced with $ref_{tissue}$ and $rec_{tissue}$. In addition to sensing distortion and artefacts, this metric measures how accurately the electric properties are reconstructed within the structure.

The Dice similarity coefficient describes spatial overlap, and is given by [53]

(9) $$\mathrm{Dice}\left(ref_{mask},\;rec_{mask}\right)=\frac{\left(ref_{mask}\cap^{\;}rec_{mask}\right)}{\frac{1}{2}\left(\left|ref_{mask}\right|+\left|rec_{mask}\right|\right)}=\frac{2\left|ref_{mask}\cap^{\;}rec_{mask}\right|}{\left|ref_{mask}\right|+\left|rec_{mask}\right|}$$

where |∙| is the cardinality of non-zero model elements within a mask.

The fourth metric assesses the proportion of malignant tissue correctly reconstructed within the tumor region (or ratio of tumor detected—RD). This is measured with [16]

(10) $$\mathrm{RD}\left(ref_{mask},\;rec_{mask}\right)=\frac{\left(ref_{mask}\cap^{\;}rec_{mask}\right)}{\left|ref_{mask}\right|}$$

where $\left|ref_{mask}\cap^{\;}rec_{mask}\right|\;$denotes taking the cardinality of non-zero model elements that are in both the reference and reconstructed masks. Values close to zero imply that the algorithm is insensitive to malignant tissue, as a very small proportion of the lesion is reconstructed within the tumor region. Conversely, values close to 1 imply that the reconstruction algorithm is sensitive to malignant tissue, as most of the malignant tissue is reconstructed within the tumor region.

The final metric is artefact rejection (AR), which measures the proportion of tissue incorrectly reconstructed as malignant tissue outside the tumor region. AR is given by [16],

(11) $$\mathrm{AR}\left(ref_{mask},\;rec_{mask}\right)=1-\frac{\left|rec_{mask}\right|-\left(ref_{mask}\cap^{\;}rec_{mask}\right)}{\left|ref_{mask}\right|}.$$

A small value of AR indicates that a large proportion of tissue has been incorrectly reconstructed as malignant tissue outside the tumor region. Conversely, values close to 1 imply that only a small proportion of the malignant tissue is reconstructed outside the tumor region. The metrics given by Equations (8), (10) and (11) are described in more detail in [16,17].

The evaluation metrics given by Equations (9)–(11) are based on the region overlap between the reference and reconstructed segmentation masks. Theses metrics are relatively insensitive to under or over estimation of the tumor region [54], so they may not be appropriate for evaluating shape fidelity. Hence, a distance-based evaluation metric referred to as the Hausdorff distance ($H_{A}$) described and analyzed in [54] provides an alternative perspective. With this measure, points extracted from the interfaces (or edges) of the reconstructed and reference masks are denoted as $rec=\left\{a_{1},a_{2},\;\ldots ,a_{Na}\right\}$ and $ref=\left\{b_{1},b_{2},\;\ldots ,b_{Nb}\right\}$, respectively. Accordingly, the Hausdorff distance evaluates how closely the shape of the reconstructed mask matches the shape of the reference mask. A variant of the Hausdorff distance between rec to ref, referred to as the average Hausdorff distance, is used for this study and is given by [55]

(12) $$H_{A}\left(ref,rec\right)=\max\left\{h\left(rec,ref\right),h\left(ref,rec\right)\right\}.$$

where

(13) $$h\left(ref,rec\;\right)=\frac{1}{N_{a}}\sum_{a\in rec}\left\{\underset{b\in ref}{\min}\|a-b\|\right\}.$$

As a pre-processing step suggested by [56], prior to computing Equation (12), the points are translated such that the center of the region enclosed by the corresponding closed contour is at the origin.

To complement the quantitative measures, qualitative assessment of images is enhanced by constructing contours from the edge points used to evaluate the average Hausdorff distances. Then, the contours are superimposed onto the forward model and reconstructed masks.

## 3. Results and Discussion

Three general case studies are used to demonstrate the utility of the proposed image analysis framework. For the first set of cases presented in Section 3.1, the forward model used to generate the numerical electromagnetic data for the study remains the same. Therefore, the shape, size, density, and tissue distribution of the breast is constant, but the degree of structural detail of the prior information (i.e., the regularization) used by the FEM-CSI algorithm varies. This leads to reconstructed images having a wide variety of image quality. The segmentation and application of metrics is shown to provide quantitative evaluation of the impact that the degree of structural detail of prior information has on image quality.

For the second set of cases that is presented in Section 3.2, the forward model used to generate the numerical data varies, but the degree of prior information used by the FEM-CSI algorithm is kept constant. Image quality is impacted primarily due to the differences in the shape, size, density, and tissue distribution of the breast being imaged, not the prior information. This demonstrates that the segmentation technique and the quantitative assessment leads to consistent results across breasts with a variety of shapes and tissue distributions.

Finally, in Section 3.3, tumor tracking cases demonstrate the potential for using the segmentation algorithm to extract clinically useful information.

### 3.1. Varying Structural Detail in Prior Information

**Figure 5:**
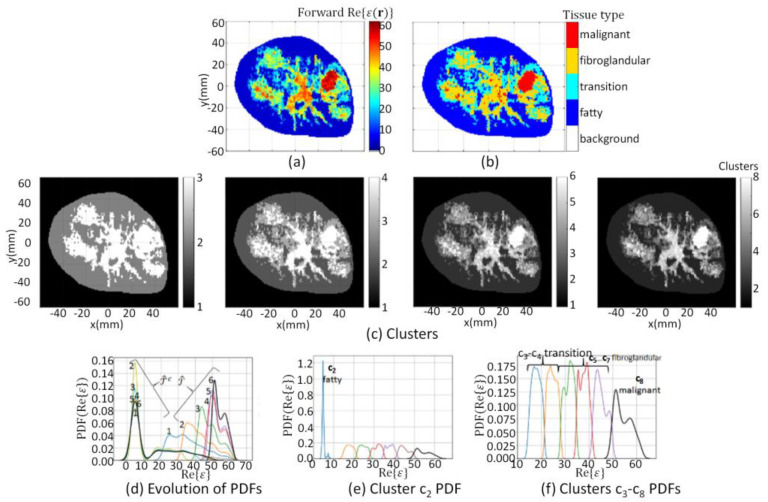
Model 1 forward model segmentation results. (**a**) ℛ extracted from forward model; (**c**) Evolution of clusters at *k* = 3, 4, 6, and 8; (**d**) Evolution of PDF over data within ${\hat{T}}^{c}$ and $\hat{T}$ where numbers indicate iteration; (**e**) PDF over data within cluster $c_{2}$, and (**f**) clusters $c_{3}$ (blue line) to $c_{8}$ (black line). Cluster $c_{2}$ corresponds to fatty tissue, $c_{3}-c_{4}$ corresponds to transition tissue, $c_{5}-c_{7}$ fibroglandular tissues, and $c_{8}$ corresponds to malignant tissue, which are mapped to segmentation masks leading to tissue type image (**b**).

The electromagnetic model (model 1) that is used for the first set of cases is a heterogeneously scattered breast constructed from an MRI slice [40]. The segmentation algorithm is applied to the real component of the complex permittivity of the forward model. The boundary, ∂𝒟, is set to the interface between the immersion medium and the skin surface. The boundary of the region of interest ∂ℛ is formed by uniformly contracting ∂𝒟 inward towards the center of the model by 3.5 mm. Mask, $mask_{ℛ},$ is formed from the region bound by ∂ℛ using Equation (3), and is applied to the forward model to recover data ℛ with Equation (4). Figure 5a shows ℛ extracted from the forward model of model 1. The same procedure is used to recover ℛ over $mask_{ℛ}\;$for the remainder of cases in this study.

The segmentation algorithm is applied to ℬ (where $ℬ=ℛ\cup^{\;}{ℛ}^{c}$) and converges after six iterations, leading to ℬ being partitioned into eight disjoint clusters. The union of clusters $c_{2}-c_{max\left\{k\right\}-1}$ form$\;{\hat{T}}^{c}$, while $c_{max\left\{k\right\}}$ form$s\;\hat{T}.$ The PDF over data within $\hat{T}$ and ${\hat{T}}^{c}$ after each iteration is shown in Figure 5d, demonstrating the convergence that terminates the segmentation process. Individual PDFs over data within each cluster $c_{2}-c_{8}$ are shown in Figure 5e,f. Finally, clusters are mapped to segmentation masks and associated tissue types using Equation (7), resulting in the segmented image shown in Figure 5b. The forward model segmentation results are used as a reference and are compared with the segmentation results of the corresponding reconstructed images.

Numerical electromagnetic data are generated with the model 1 forward model. For the first case (3.1a), detailed patient-specific prior information is provided. Accordingly, the inhomogeneous background *ϵ_b_*(**r**) in (1) emulates the structural information that would be recovered from an MRI image. This process is described in more detail in [16].

**Figure 6:**
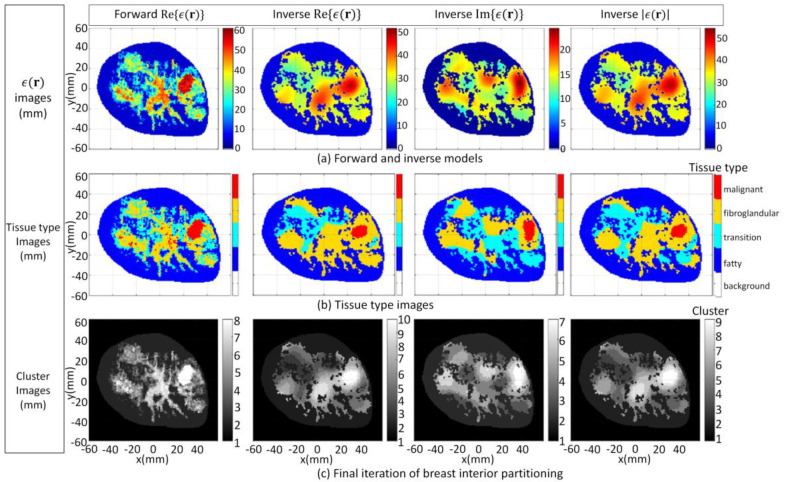
Case 3.1a forward model and reconstruction results when algorithm applied to model 1 data and *ϵ_b_*(**r**) is set to detailed internal structure (**a**); Tissue type images (**b**); Final iteration of segmentation algorithm (**c**).

The FEM–CSI algorithm reconstructs the contrast profile χ(r); then, Equations (1) and (2) are employed to recover a list of images from (**r**), given by Re{*ϵ*(**r**)}, Im{*ϵ*(**r**)}, and |*ϵ*(**r**)|. These images are shown Figure 6a. The tissue type and cluster images formed when the segmentation algorithm is applied are shown in Figure 6b,c, respectively. More detailed results in a format similar to Figure 5 showing the evolution of the PDF over data within $\hat{T}$ and ${\hat{T}}^{c}$ and the clusters after each iteration are furnished by Supplementary Materials Figures S1–S10. Moreover, the detailed results for all of the cases examined in Section 3.1 and video demonstrations are also available from the repository described in [52].

**Figure 7:**
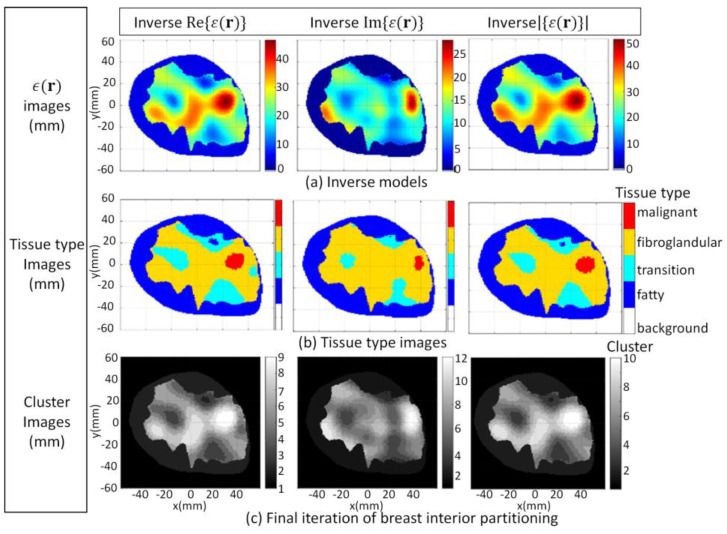
Case 3.1b reconstruction results when algorithm applied to model 1 data and *ϵ_b_*(**r**) is set to structural information related to skin, fat, and glandular regions extracted by radar-based technique (**a**); Tissue type images (**b**); Final iteration of segmentation algorithm (**c**).

For the second case (3.1b), the inhomogeneous background *ϵ_b_*(**r**) in Equation (1) is set to information extracted from radar-based techniques described in [16,57,58,59] and has less detail relative to the first case. Specifically, structural information related to the skin, fat, and glandular regions is provided along with estimates of the mean dielectric properties over these regions. The corresponding images reconstructed by the FEM-CSI algorithm are shown in Figure 7a and exhibit a lower degree of quality relative to the first case. The tissue type and cluster images are shown in Figure 7b,c, respectively.

**Figure 8:**
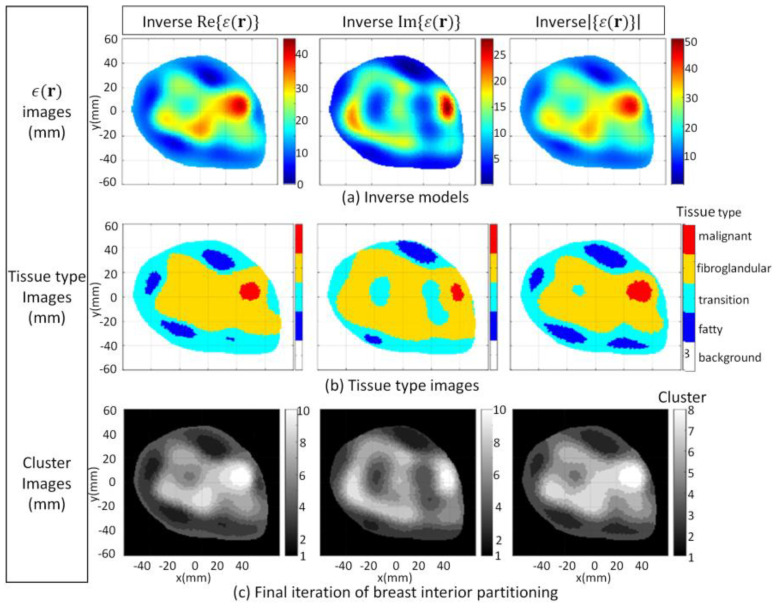
Case 3.1c reconstruction results when algorithm applied to model 1 data and *ϵ_b_*(**r**) is set to structural information related to skin region extracted by radar-based technique (**a**); Tissue type images (**b**); Final iteration of segmentation algorithm (**c**).

For the third and final case (3.1c), the inhomogeneous background *ϵ_b_*(**r**) in Equation (1) incorporates structural information related to the skin region along with a homogenous breast interior with complex dielectric properties estimated with [16,57,58,59]. The reconstructed results shown in Figure 8a exhibit the lowest degree of quality of the three cases studied in this section, and they are the most challenging to segment. The tissue type mapping and cluster images are shown in Figure 8b,c, respectively.

**Figure 9:**
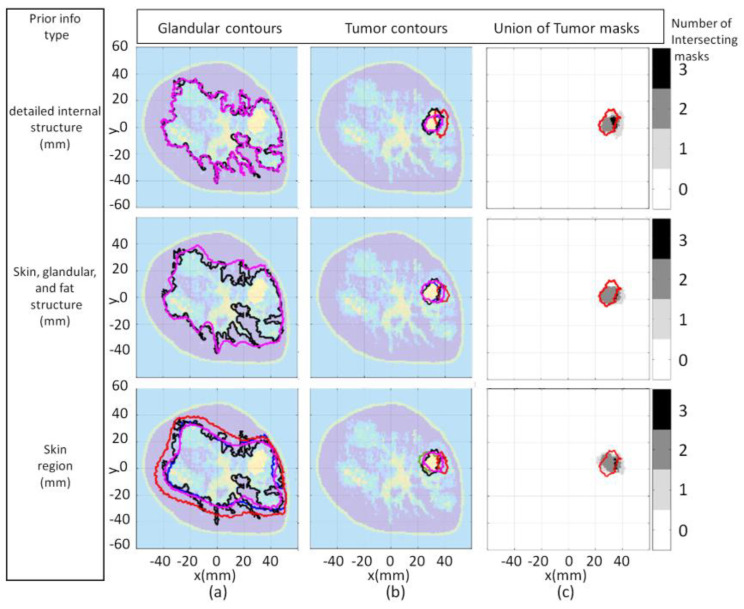
Model 1 qualitative image analysis of reconstructed images formed using various prior information detail. Glandular mask contours (**a**), and tumor mask contours (**b**) with contours extracted from forward model (black-line), reconstructed Re{ϵ(r)} (blue-line), Im{ϵ(r)} (red-line), and |ϵ(r)| (pink-line). Forward model contour (red-line) superimposed onto union of reconstructed tumor masks (**c**).

The consistency of the proposed approach becomes particularly useful when segmenting images for which interfaces that delineate tissue types are blurred or are incorrectly located. This is evident for all three cases when segmenting the malignant from fibroglandular tissue and when segmenting the fibroglandular tissues from the breast interior for the third case. In addition to blurred interfaces, differences in electrical properties reconstructed that depends on the degree of structural detail of the prior information used by the FEM-CSI algorithm is also observed for the three cases. Regardless of these challenges, the proposed segmentation methodology gives reasonable estimates of glandular and tumor regions in all reconstructions. The qualitative image analysis is shown for all three cases in Figure 9. The regional and distance-based metrics are applied to the glandular and tumor regions, leading to the quantitative results shown in Table 1 and Table 2, respectively.

The effectiveness of the metrics incorporating segmentation results is evident from the results shown in Table 1 and Table 2. As expected, the values of the metrics demonstrate that reducing the structural detail in the prior information leads to a degradation of reconstruction of the glandular structure. However, reducing this structural detail also impacts the quality of the reconstruction of the tumor region in a more complicated manner. For this set of examples, the specificity (implied by value of AR) degrades and the sensitivity improves (implied by value of RD) with decreasing amounts of structural prior information. Furthermore, each component of the reconstruction is impacted differently. Namely, the quality of the imaginary component in terms of sensitivity (RD) and tumor shape (H_A_) benefits from a greater detail of prior structural information relative to the real component. These examples demonstrate the utility of having a framework that effectively provides a quantitative assessment of regions that contain a specific tissue. In particular, the regional and distance metrics provide valuable insight into a complex issue such as the evaluation of the impact that the degree of structural detail of prior information has on image quality.

**Figure 10:**
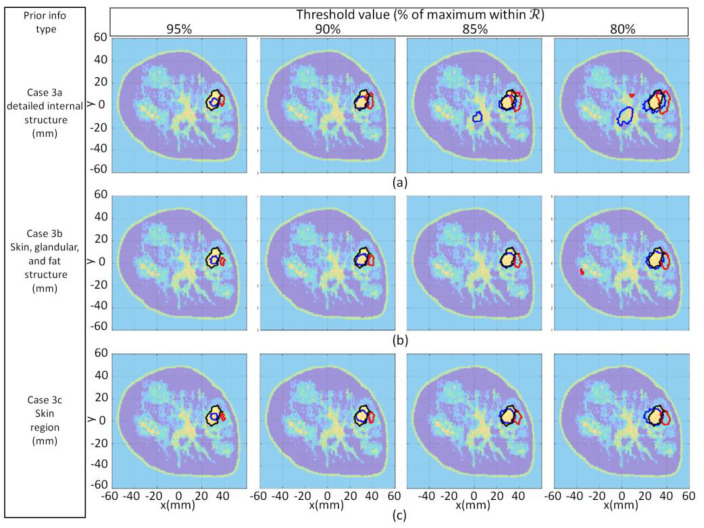
Model 1 qualitative image analysis of reconstruction images using various threshold values applied to cases 3.1a (**a**), 3.1b (**b**), and 3.1c (**c**). For each case, contours associated with tumor masks from forward model, reconstructed Re{*ϵ*(**r**)}, and Im{*ϵ*(**r**)} shown with black, blue, and red lines, respectively, superimposed onto forward model.

A key motivation for developing the proposed segmentation methodology is to resolve the challenges that arise when using thresholding techniques. The challenges are demonstrated by applying the thresholding technique implemented by the studies described in [16,17] to the reconstructed images in this section. Specifically, threshold values are set to 95%, 90%, 85% and 80% of the maximum reconstructed value within the breast interior. In Figure 10, the black contour extracted from the forward model serves as a ground truth for comparison with the thresholded tumor contours. Likewise, metrics are applied to the reference and reconstructed tumor masks resulting from thresholding and are presented in Table 3.

The results shown in Figure 10 and Table 3 demonstrate the challenge of determining an appropriate threshold value to use with the threshold-based segmentation technique. Namely, adjustments of the threshold values demonstrate the trade-off between sensitivity and specificity that classification problems experience when using a methodology that depends on a fixed threshold value. For example, setting the segmentation threshold value for malignant tissue too low (e.g., 80%) leads to an improvement in sensitivity (i.e., high RD value) at the expense of the deterioration of the specificity (i.e., decrease in AR). This occurs because model elements that are within the fibroglandular structure are incorrectly attributed to malignant tissue. Likewise, setting the threshold value too high (e.g., 95%) impacts sensitivity by incorrectly assigning reconstructed tissue to fibroglandular tissue when it is, in fact, malignant tissue. Accordingly, the choice of what value of threshold to use is not obvious and, to complicate matters, it has been observed that the maximum value of the reconstructed tissue using FEM-CSI depends on the number of iterations.

In contrast, the proposed technique does not rely on assumed dielectric property values of the reconstructed tissues. Moreover, the proposed iterative approach does not require the number of clusters to be pre-selected, as the unsupervised machine learning technique is reinforced with hypothesis testing and statistical inference to automatically determine the number of clusters.

The convenience of using this strategy is evident when observing the variation in the final number of clusters, as shown in the bottom row of Figure 6, Figure 7 and Figure 8. The examples demonstrate that pre-selecting the number of clusters beforehand is not practical. Furthermore, using the proposed strategy leads to a more precise and consistent approach to image analysis compared to alternative methods by automatically detecting regions of interest in the image corresponding to various tissue types or anomalies. This advantage is particularly evident when comparing the metric values in Table 2 with those in Table 3. In Table 3, there is a significant variation in the values of all metrics across all reconstruction components and test cases, depending on the threshold value used. The variation in the metric values leads to inconsistent results that contribute to unreliable quantitative assessment of reconstructed images.

It is also observed that the threshold technique requires different threshold values in order to achieve the same results as the proposed automatic segmentation method. For example, for case 3.1a, the thresholding technique requires values of approximately 90% and less than 85% to segment the real and imaginary components, respectively. Different threshold values are also needed depending on the image component and the case examined. This observation demonstrates that using the proposed technique leads to a simplification of the segmentation process that may result in improved consistency and reliability of results. Moreover, it is not necessary for the user to make a decision on a threshold value to use or to iteratively fine tune threshold values depending on the image component or reconstructed image. This observation also demonstrates the flexibility of the proposed technique and its ability to automatically adapt to a scenario (e.g., image quality).

### 3.2. Varying Breast Shape and Tissue Distribution

The second part of the study is comprised of three cases, namely breast models with different shapes and tissue distributions. The degree of prior information used by the FEM-CSI algorithm is kept constant, so image quality is impacted primarily due to the shape, size, and tissue distribution of the breast being imaged. The inhomogeneous background *ϵ_b_*(**r**) in Equation (1) is extracted from ultrasound data described in [60]. An electromagnetic model (model 3.2a) described in [40] of a heterogeneously dense breast that is constructed from an MRI slice is used for the first case.

**Figure 11:**
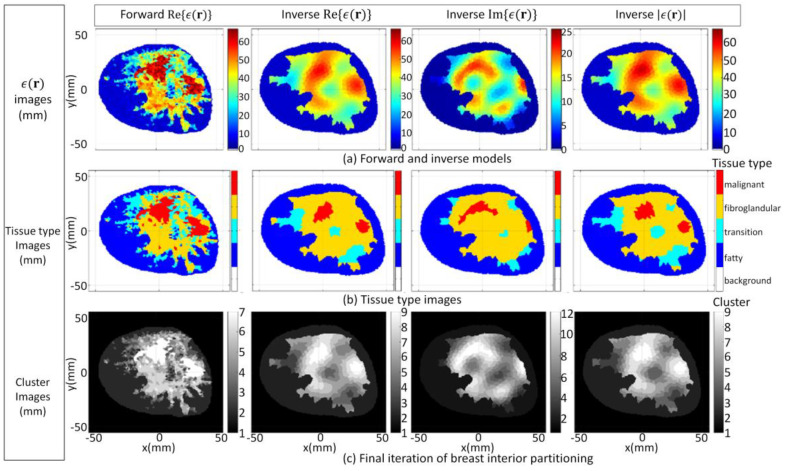
Model 3.2a forward model and reconstruction results (**a**); Tissue type images (**b**); Final iteration of segmentation algorithm (**c**).

**Figure 12:**
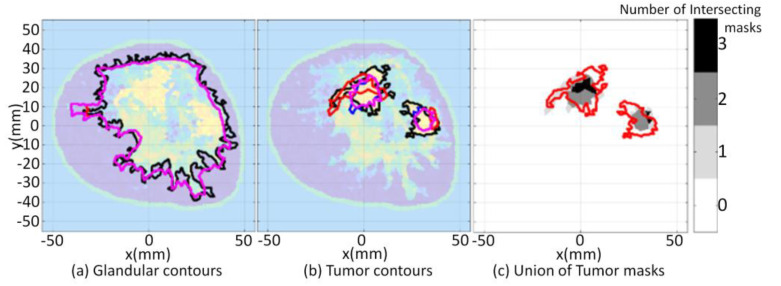
Model 3.2a qualitative image analysis. Glandular mask contours (**a**), and tumor mask contours (**b**) with contours extracted from forward model (black-line), reconstructed Re{ϵ(r)} (blue-line), Im{ϵ(r)} (red-line), and |ϵ(r)| (pink-line). Forward model contour (red line) superimposed onto union of reconstructed tumor masks (**c**).

When applied to the forward model, the segmentation algorithm converges after five iterations, leading to ℬ being partitioned into seven disjoint clusters. These clusters are mapped to masks and associated tissue types using Equation (7). The forward model segmentation results are used as a reference and are compared with the segmentation results of the corresponding reconstructed images. Numerical electromagnetic data are generated with forward model 3.2a. The FEM-CSI algorithm iteratively reconstructs the contrast profile [17] and the corresponding images, given by Re{*ϵ*(**r**)}, Im{*ϵ*(**r**)}, and |*ϵ*(**r**)|, are shown in Figure 11a. The tissue type and cluster images are shown in Figure 11b,c, respectively. The qualitative image analysis is shown in Figure 12. The regional and distance-based metrics lead to the quantitative results shown in Table 4.

**Figure 13:**
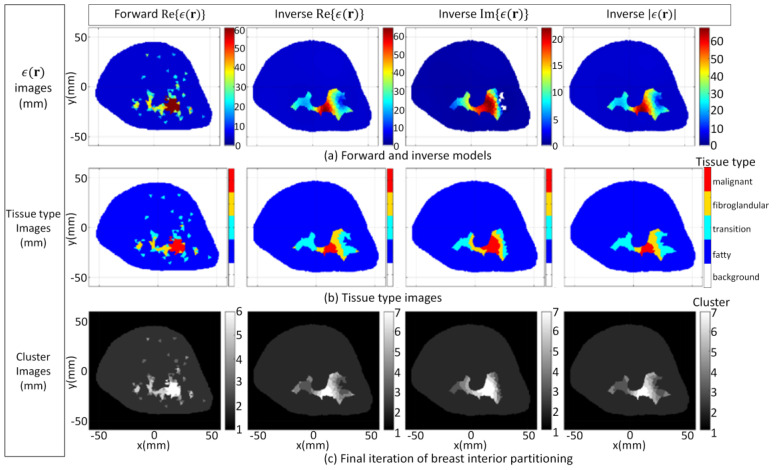
Model 3.2b forward model and reconstruction results (**a**); Tissue type images (**b**); Final iteration of segmentation algorithm (**c**).

**Figure 14:**
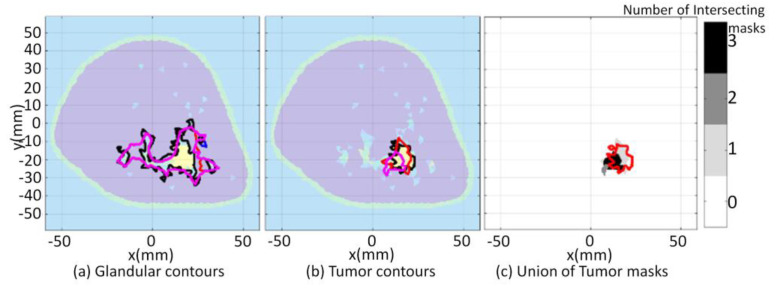
Model 3.2b qualitative image analysis. Glandular mask contours (**a**), and tumor mask contours (**b**) with contours extracted from forward model (black-line), reconstructed Re{ϵ(r)} (blue-line), Im{ϵ(r)} (red-line), and |ϵ(r)| (pink-line). Forward model contour (red line) superimposed onto union of reconstructed tumor masks (**c**).

Model 3.2b is an electromagnetic model of a fatty breast that is constructed from a sequence of MRI slices described in [30]. The segmentation algorithm is applied to the forward model and converges after four iterations. The FEM-CSI algorithm iteratively reconstructs the contrast profile [17]. Results obtained when the segmentation algorithm is applied to the forward model and the reconstructed images are shown in Figure 13. The qualitative image analysis is shown in Figure 14, while regional and distance-based metrics are summarized in Table 5.

**Figure 15:**
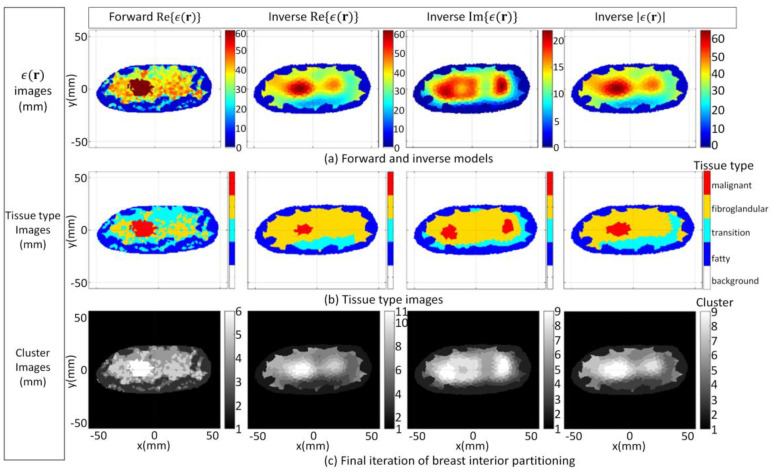
Model 3.2c forward model and reconstruction results (**a**); Tissue type images (**b**); Final iteration of segmentation algorithm (**c**).

**Figure 16:**
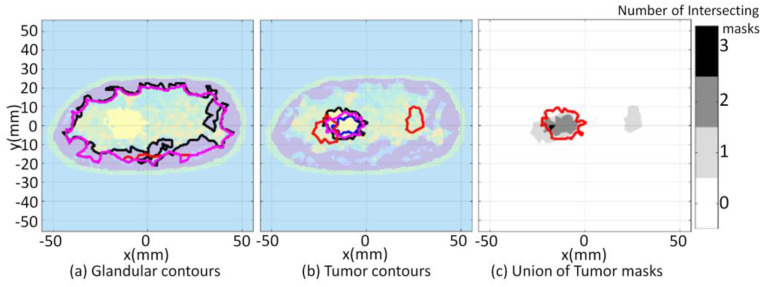
Model 3.2c qualitative image analysis. Glandular mask contours (**a**), and tumor mask contours (**b**) with contours extracted from forward model (black-line), reconstructed Re{ϵ(r)} (blue-line), Im{ϵ(r)} (red-line), and |ϵ(r)| (pink-line). Forward model contour (red line) superimposed onto union of reconstructed tumor masks (**c**).

Model 3.2c is used as the final case studied for this part of the study, and it is an electromagnetic model of a dense breast that is constructed from a sequence of MRI slices [30]. The segmentation algorithm is applied to the forward model and converges after four iterations. The FEM-CSI algorithm iteratively reconstructs the contrast profile [17]. The results obtained when the segmentation algorithm is applied to the forward model and the reconstructed images are shown in Figure 15. The qualitative image analysis is shown in Figure 16, and a summary of the regional and distance-based metrics is provided in Table 6.

For this section, the tissue distribution of each model varied, but the prior knowledge of internal structural information was kept the same. Even with considerable variation in breast density and tissue distribution between models, it was demonstrated that the segmentation algorithm is robust to these variations. As observed with the cases in Section 3.1, the final number of clusters that the algorithm converges to varies, depending on the tissue distribution of the breast and image component being segmented. Unlike thresholding segmentation techniques that require pre-selected thresholds, or an unsupervised machine learning approach such as *k-*means clustering that requires a pre-selected number of clusters, the proposed image segmentation does not require prior information. Consequently, it is not data-specific, unlike these other techniques, and it was able to reliably and consistently segment the reconstructed images into tissue types to permit the quantitative assessment of regions that contain a specific tissue.

These results also provide insight into the impact that the breast density and tissue distribution has on the performance of the FEM-CSI algorithm. Specifically, reconstruction of the real and imaginary components of the malignant tissue was effectively assessed. For the imaginary component, the metrics suggest that the reconstruction algorithm is more sensitive to malignant tissue (i.e., higher RD value) and reconstructed the tumor region more accurately (lower H_A_ value) for the fatty breast compared to the other two cases. On the other hand, for the real component, the metrics suggest that the reconstruction algorithm is equally sensitive to the malignant tissue for all three tissue distributions. However, similar to the imaginary component, the tumor region of the real component was reconstructed more accurately for the fatty breast scenario. For the dense breast, the advantages of analyzing the magnitude of the reconstructed image is evident, as there is both an improvement in sensitivity and accuracy of the tumor region that is reconstructed compared to the quality of the real and imaginary components.

Similar to the test cases studied in Section 3.1, the examples investigated in this section demonstrate the utility of having a framework that effectively provides a quantitative assessment of regions that contain a specific tissue to provide valuable insight into a complex issue. Namely, the evaluation of the impact that the tissue distribution and breast density have on image quality and the performance of the reconstruction algorithm can be effectively assessed. These insights are not necessarily revealed or as obvious with a qualitative assessment such a visual examination and image comparisons.

The test cases also demonstrate the practical utility of mapping clusters to distinct tissue types. The tissue mapped images may be used to assist with image interpretation and to more readily identify anomalies.

### 3.3. Tumor Tracking

The contrast in dielectric properties between healthy and malignant tissues reported in the large-scale studies [2,3,4,5,6,7] may be exploited with microwave imaging in order to image malignant tissue. This is supported with clinical studies described in [10,12,24,25] that demonstrate the utility of microwave tomography for breast screening and therapy monitoring. Consequently, the final part of the study is comprised of two tumor tracking examples to demonstrate that the segmentation technique may assist with extracting clinically useful information. Similar to the second part of the study described in Section 3.2, the degree of structural detail of the prior information used by the FEM-CSI algorithm is the same for each case. For both cases, the inhomogeneous background *ϵ_b_*(**r**) in (1) is set to information extracted from the radar-based technique described in [16,57,58,59]. Model 1, which is also used in Section 3.1, is the forward model used to generate the numerical electromagnetic data.

**Figure 17:**
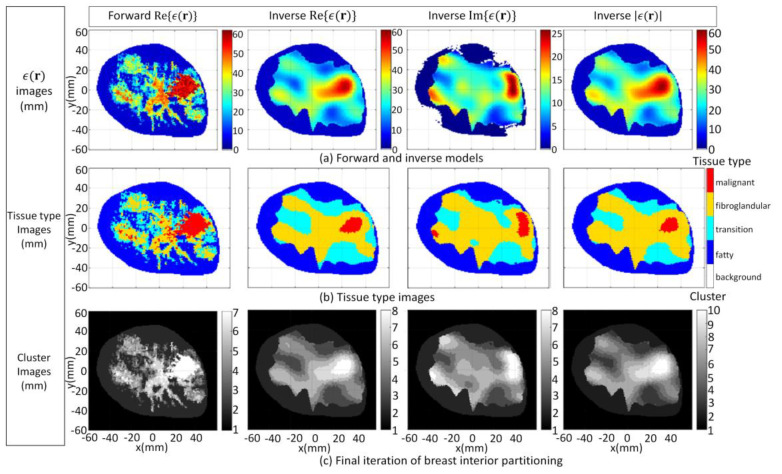
Model 1 forward model with large tumor embedded in fibroglandular tissues and reconstruction results (**a**); Tissue type images (**b**); Final iteration of segmentation algorithm (**c**).

For the first case (3.3a), a large tumor region is present in the forward model, as shown in Figure 17. The segmentation algorithm is applied to the forward model and converges after five iterations, so ℬ is partitioned into seven disjoint clusters. These clusters are mapped to segmentation masks and associated tissue types using Equation (7). The forward model segmentation results are used as a reference and are compared with the segmentation results of the corresponding reconstructed images.

The FEM-CSI algorithm iteratively reconstructs the contrast profile [17]. The corresponding images are shown in Figure 17a. The tissue type and cluster images are shown in Figure 17b,c, respectively.

For the second case (3.3b), the size of the tumor region is reduced, but its location within the forward model is approximately the same as the first case. The results when the segmentation algorithm is applied to the forward model and the reconstructed images are shown in Figure 7 (Section 3.1).

**Figure 18:**
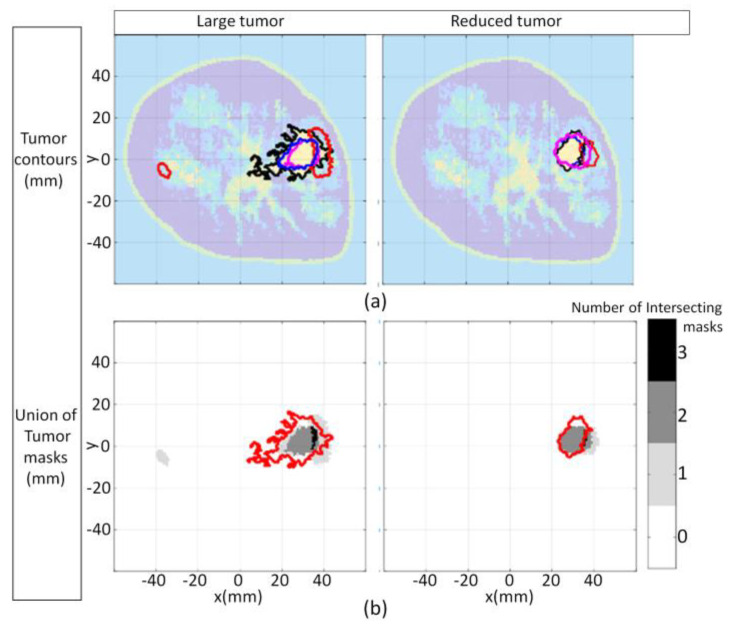
Model 1 tumor tracking qualitative image analysis. Contours for large tumor and reduced tumor cases (**a**) with contours extracted from forward model (black line), reconstructed Re{ϵ(r)} (blue line), Im{ϵ(r)} (red line), and |ϵ(r)| (pink line). Forward model contour (red line) superimposed onto union of masks formed with malignant tissue reconstructed from FEM-CSI Re{ϵ(r)}, Im{ϵ(r)}, |ϵ(r)| (**b**).

The qualitative image analysis is shown for each case in Figure 18. The region and distance-based metrics are applied to the reference and reconstructed masks of the tumor regions, leading to the quantitative results shown in Table 7.

The potential for using the algorithm to provide clinically useful information is demonstrated with this set of tumor tracking examples. Microwave tomography typically produces lower resolution images than clinical imaging methods such as X-ray. Hence, segmenting medical images formed with microwave tomography for tumor tracking examples can be challenging as the interfaces that delineate tissue types may be blurred. This is particularly challenging when malignant tissue is embedded in glandular tissue. Contributing to the challenge is the possibility that there may be a great deal of inhomogeneity amongst the glandular tissue. Regardless of these challenges, the proposed segmentation procedure demonstrated the ability to delineate the reconstructed tissue from the glandular tissue.

Once the tissue regions are extracted, metrics are applied for quantitative analysis in order to assess the results. The metrics shown in Table 7 infer that for the large tumor reconstruction scenario, the algorithm is less sensitive but has a higher specificity to the malignant tissue relative to the reduced tumor scenario. The values of the average Hausdorff distance shown in Table 7 indicate that the reconstruction algorithm did not reconstruct the shape of the malignant region as accurately compared to the reduced tumor scenario. The metrics collectively suggest that there is inadequate information furnished from the images to make a judgement with respect to whether a significant reduction in the size of the malignant region has occurred (in response to some treatment, for example).

Similar to the test cases examined in the previous sections, this set of cases demonstrate the practical convenience of mapping clusters to distinct tissue types. The tissue mapped images may be used to assist with image interpretation and to more readily make inferences on the location of the malignant tissue within the glandular structure. This example also demonstrates the utility of providing a framework for assessing the performance of the reconstruction algorithm. For example, the metrics may be used to inform researchers with regard to adjustments to the reconstruction algorithm or measurement system parameters such as an increase in the number of sensors to improve the sensitivity and overall performance of the reconstruction algorithm.

## 4. Conclusions

A medical image segmentation technique has been presented that partitions microwave breast images into regions of interest corresponding to distinct tissue types in order to facilitate the evaluation of image quality. A key advantage for using the algorithm over other approaches is that it supports a quantitative analysis of microwave images without prior assumptions such as knowledge of the expected dielectric property values that characterize each tissue type. Unlike supervised machine learning approaches that require copious amounts of data to effectively train a model, it can be used for scenarios where there is a scarcity of data. It also addresses a significant difficulty encountered by many unsupervised machine learning approaches in that it does not require a predetermined number of clusters to partition the image. The proposed technique is not data-specific, as it was able to segment a variety of images with different image quality. Moreover, it was able to reliably and consistently segment images derived from breasts with various tissue distributions and densities into tissue types to permit quantitative assessment of regions that contain a specific tissue.

The segmentation into tissue types leads to the decomposition of the breast interior into disjoint tissue masks. An array of region and distance-based metrics were applied to compare masks extracted from reconstructed images and ground truth models. The quantitative results revealed the accuracy with which the geometric and dielectric properties are reconstructed. The incorporation of the segmentation results into an evaluation framework with metrics was demonstrated and effectively furnished quantitative assessment of tissue-specific regions. The examples demonstrated the utility of having this framework to provide valuable insight into a complex issue. Namely, the impact that changes in tissue distribution and breast density have on image quality and the performance of the reconstruction algorithm can be effectively assessed. These insights are not necessarily revealed or as obvious with a qualitative assessment such a visual examination and image comparisons.

It is anticipated that this framework may also be applied to the analysis of the data acquisition environment to quantify changes in image quality to inform researchers on the number and location of sensors, the incident field frequency, measurement chamber design, and the orientation of the receivers relative to the data acquisition surface. For this study, the numerical breast models were used for the forward model and furnished the reference regions to compare with the tissues segmented from the image. However, when using clinical data, the reference model may be the patient at a previous point in time to quantify how a region changed over time in response to a treatment. The reference model for clinical or experimental data may also be an inverse model obtained with variations on the same algorithm or a different reconstruction algorithm (comparing the FEM-CSI inverse solver with the Distorted Born iterative method, for example).

In addition to facilitating a quantitative analysis of images, the tissue masks facilitate supplying qualitative information to assist in the interpretation of the microwave images. This qualitative information is augmented with images showing the location of estimated tissue interfaces that provide a visual means to quickly interpret an image or the performance of an inversion algorithm.

More broadly, the presented technique provides a general framework that may be applied to an extensive range of medical imaging modalities. This may be particularly useful for developing modalities for which users do not have much experience with the reconstructed images, as well as when there is scarcity of data available for supervised learning. Initial investigations into the application of the technique to ultrasound images has assisted with studies reported in [17,60]. The diverse range of potential applications that may implement the presented image analysis technique also includes liquid biopsy analysis [61,62,63].

Future work includes integrating this segmentation approach with performance metrics (e.g., [16,17,39,60]), and composite tissue-type and probability images [64].

## Figures and Tables

**Table 1:** Model 1: Glandular region metrics—varying degree of prior information.

Case	Metric	Real	Imaginary	Magnitude
	Fidelity	0.95	0.95	0.95
3.1a (detailed internal structure)	Dice	0.95	0.95	0.95
	xcorrDiel	0.91	0.89	0.91
	H_A_	0.66	0.66	0.66
	Fidelity	0.85	0.85	0.85
3.1b (regional internal structure)	Dice	0.85	0.85	0.85
	xcorrDiel	0.85	0.82	0.85
	H_A_	5.68	5.68	5.68
	Fidelity	0.85	0.81	0.86
3.1c (skin region)	Dice	0.85	0.79	0.86
	xcorrDiel	0.83	0.75	0.83
	H_A_	4.39	7.09	4.06

**Table 2:** Model 1: Tumor region metrics—varying degree of prior information.

Case	Metric	Real	Imaginary	Magnitude
	RD	0.63	0.20	0.60
3.1a (detailed internal structure)	AR	0.95	0.37	0.87
	Dice	0.75	0.22	0.69
	H_A_	1.56	1.63	1.44
	RD	0.77	0.01	0.69
3.1b (regional internal structure)	AR	0.88	0.65	0.78
	Dice	0.82	0.01	0.73
	H_A_	1.20	2.73	1.32
	RD	0.71	0.05	0.86
3.1c (skin region)	AR	0.87	0.60	0.61
	Dice	0.77	0.06	0.76
	H_A_	1.44	2.42	1.49

**Table 3:** Model 1 tumor region metrics: tumor region extracted with threshold technique using various values of threshold.

Case	Metric	95%	90%	85%	80%
	RD	0.26	0.64	0.76	0.90
3.1a (detailed internal structure)	AR	0.98	0.94	0.39	−0.62
Real component	Dice	0.41	0.75	0.64	0.51
	H_A_	3.46	1.47	1.28	1.78
	RD	0.05	0.11	0.23	0.33
3.1a (detailed internal structure)	AR	0.79	0.65	0.44	0.16
Imaginary component	Dice	0.07	0.15	0.26	0.31
	H_A_	3.35	2.28	1.44	1.48
	RD	0.26	0.53	0.72	0.83
3.1b (regional internal structure)	AR	1.00	0.98	0.90	0.66
Real component	Dice	0.40	0.68	0.79	0.76
	H_A_	3.53	1.95	1.34	1.16
	RD	0.00	0.00	0.02	0.05
3.1b (regional internal structure)	AR	0.86	0.74	0.61	0.41
Imaginary component	Dice	0.00	0.00	0.03	0.61
	H_A_	4.35	3.33	2.39	1.83
	RD	0.21	0.52	0.73	0.82
3.1c (skin region)	AR	1.00	0.99	0.84	0.61
Real component	Dice	0.35	0.68	0.78	0.74
	H_A_	4.01	2.13	1.32	1.50
	RD	0.00	0.00	0.03	0.06
3.1c (skin region)	AR	0.88	0.76	0.65	0.54
Imaginary component	Dice	0.00	0.01	0.04	0.08
	H_A_	4.47	3.41	2,63	2.11

**Table 4:** Model 3.2a quantitative results.

Region	Metric	Real	Imaginary	Magnitude
	Fidelity	0.90	0.90	0.90
Glandular	Dice	0.90	0.90	0.90
	xcorrDiel	0.91	0.88	0.91
	H_A_	1.66	1.64	1.66
	RD	0.44	0.35	0.50
Tumor 1	AR	0.92	0.78	0.96
	Dice	0.58	0.45	0.65
	H_A_	3.80	3.66	3.71
	RD	0.40	0.09	0.36
Tumor 2	AR	0.94	0.93	0.94
	Dice	0.55	0.15	0.51
	H_A_	2.85	4.52	3.39

**Table 5:** Model 3.2b quantitative results.

Region	Metric	Real	Imaginary	Magnitude
	Fidelity	0.61	0.65	0.62
Glandular	Dice	0.59	0.64	0.60
	xcorrDiel	0.72	0.79	0.72
	H_A_	3.34	2.54	3.29
	RD	0.34	0.76	0.34
Tumor	AR	0.71	0.61	0.71
	Dice	0.41	0.71	0.41
	H_A_	1.62	1.10	1.62

**Table 6:** Model 3.2c quantitative results.

Region	Metric	Real	Imaginary	Magnitude
	Fidelity	0.87	0.88	0.87
Glandular	Dice	0.87	0.87	0.87
	xcorrDiel	0.92	0.92	0.92
	H_A_	2.13	2.16	2.13
	RD	0.37	0.16	0.69
Tumor	AR	1.00	0.65	0.95
	Dice	0.54	0.21	0.79
	H_A_	2.96	2.56	1.36

**Table 7:** Model 1 tumor tracking quantitative results.

Case	Metric	Real	Imaginary	Magnitude
	RD	0.46	0.17	0.30
3.3a—Large tumor	AR	1.00	0.76	1.00
	Dice	0.63	0.24	0.46
	H_A_	4.90	6.67	5.71
	RD	0.77	0.01	0.69
3.3b—Reduced tumor	AR	0.88	0.65	0.78
	Dice	0.82	0.01	0.73
	H_A_	1.20	2.73	1.32

## Data Availability

Publically available datasets related to the scattered density, and heterogeneously dense categorized breasts were analyzed in this study. These data can be found here: https://github.com/djkurran/MWSegEval/testData. Use [40] when citing these data. Moreover, publically available datasets related to the fatty and extremely dense categorized breasts analyzed in this study for Case 3.2 are available here: Omer, M., Fear, E. Anthropomorphic breast model repository for research and development of microwave breast imaging technologies. *Sci Data* **5**, 180257 (2018). https://doi.org/10.1038/sdata.2018.257. Use [65] when citing these model data. The novel computer code and software developed by the authors that integrates the unsupervised machine learning and thresholding segmentation techniques into an image processing toolbox are available in the publically available repository: https://github.com/djkurran/MWSegEval. A wiki page associated with this repository hosts a detailed on-line manual for the toolbox. Use [22] when citing the toolbox.

## Supplementary Materials

The following are available online at https://www.mdpi.com/2313-433X/7/1/5/s1, Detailed results for all cases presented in Section 3 including the clusters after each iteration and the evolution of the PDF of the data over $\hat{T}$ and ${\hat{T}}^{c}$ are available online at https://github.com/djkurran/Segmentation-unsupervised-machine-learning [52]. A list of figures available in the repository is as follows: Figure S1: Model 1 forward model segmentation results, Figure S2: Case 3.1a Segmentation results of reconstruction derived from detailed internal structure prior—Real component, Figure S3 Case 3.1a Segmentation results of reconstruction derived from detailed internal structure prior—Imaginary component, Figure S4 Case 3.1a Segmentation results of reconstruction derived from detailed internal structure prior—Magnitude, Figure S5 Case 3.1b Segmentation results of reconstruction derived from regional internal structure prior—Real component, Figure S6 Case 3.1b Segmentation results of reconstruction derived from regional internal structure prior—Imaginary component, Figure S7 Case 3.1b Segmentation results of reconstruction derived from regional internal structure prior—Magnitude, Figure S8 Case 3.1c Segmentation results of reconstruction derived from skin region prior—Real component, Figure S9 Case 3.1c Segmentation results of reconstruction derived from skin region prior—Imaginary component, Figure S10 Case 3.1c Segmentation results of reconstruction derived from skin region prior—Magnitude.
