# Supplementary material for: Evaluating Performance of Microwave Image Reconstruction Algorithms: Extracting Tissue Types with Segmentation Using Machine Learning
Source: J Imaging. 2021 Jan 7;7(1):5. doi: 10.3390/jimaging7010005 (PMC8321253; doi:10.3390/jimaging7010005)
Supplement: Supplementary file 1 [file jimaging-07-00005-s001.pdf]

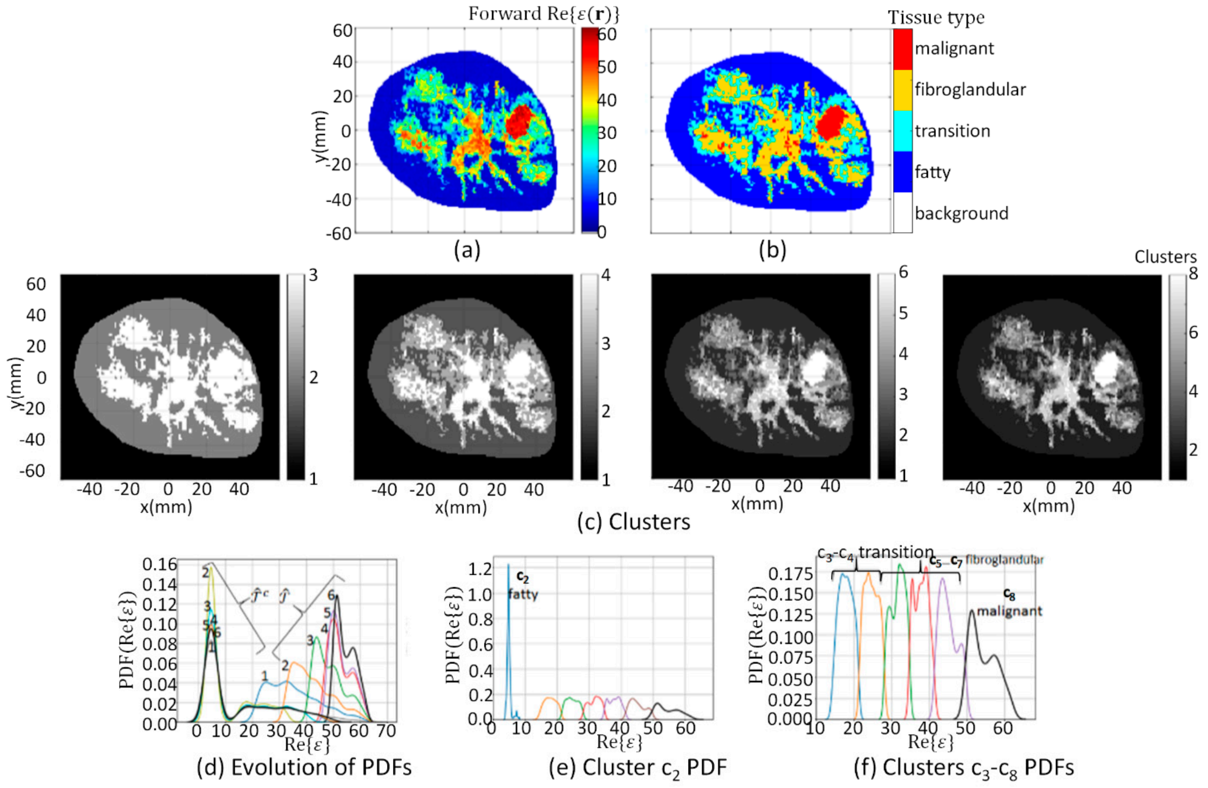

Figure S1. Model 1 forward model segmentation results. (**a**) Region $\mathcal{R}$ extracted from forward model; (**c**) Evolution of clusters at k = 3, 4, 6, and 8; (**d**) Evolution of probability density function (PDF) over data within $\hat{\mathcal{T}}^c$and $\hat{\mathcal{T}}$ where numbers indicate iteration; (**e**) PDF over data within cluster c_2, and (**f**) clusters c_3 (blue line) to c_8 (black line). Cluster c_2 corresponds to fatty tissue, $c_3 - c_4$ corresponds to transition tissue, $c_5 - c_7$ fibroglandular tissues, and c_8 corresponds to malignant tissue, which are mapped to segmentation masks leading to tissue type image (**b**).

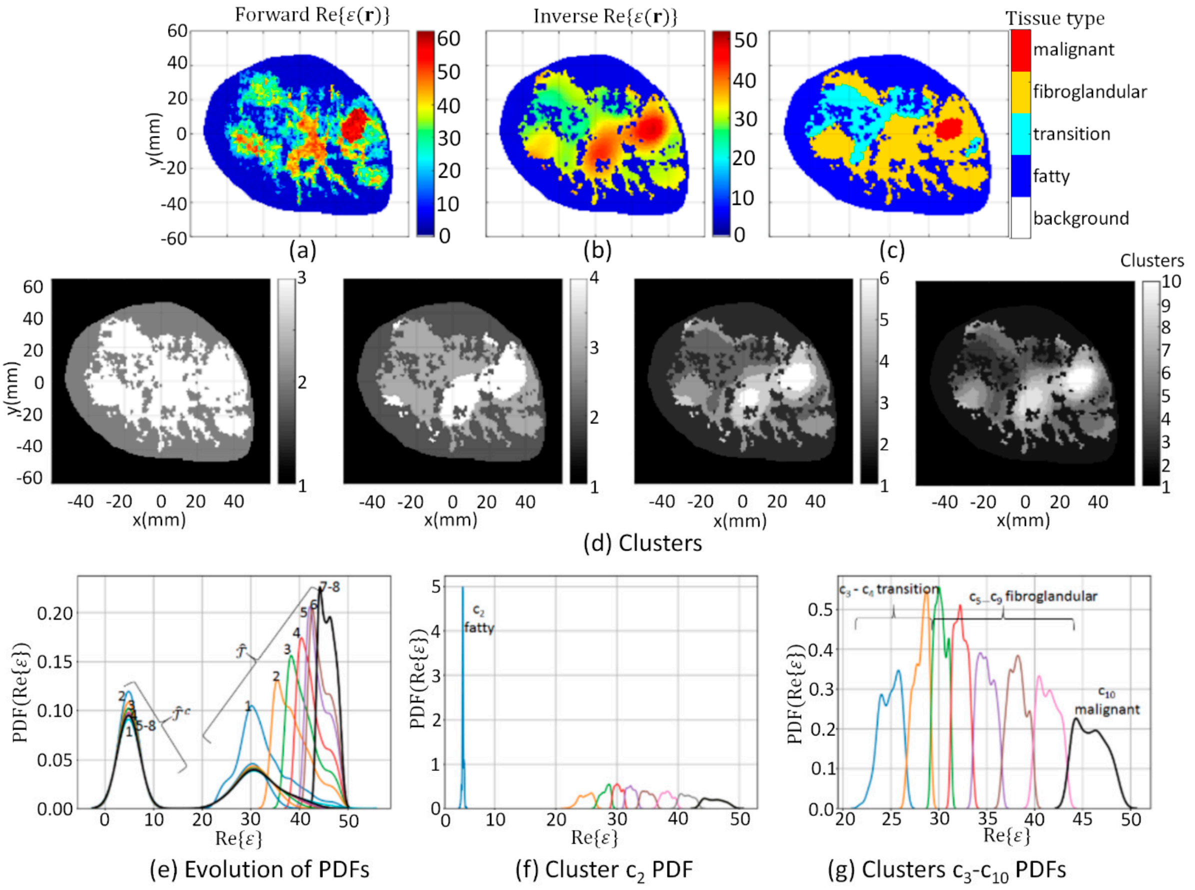

Figure S2. Case 3.1a Segmentation results of reconstruction derived from detailed internal structure prior-Real component. (**a**) Region $\mathcal{R}$ extracted from forward model; (b) Region $\mathcal{R}$ extracted from real component of inverse model; (d) Evolution of clusters at k = 3, 4, 6, and 10; (e) Evolution of PDF over data within $\hat{\mathcal{T}}^c$and $\hat{\mathcal{T}}$ where numbers indicate iteration; (f) PDF over data within cluster c_2, and (**g**) clusters c_3 (blue line) to c_{10} (black line). Clusters $c_3 - c_4$ corresponds to transition tissue, $c_5 - c_9$ fibroglandular tissues, and c_{10} corresponds to malignant tissue, which are mapped to segmentation masks leading to tissue type image (**b**).

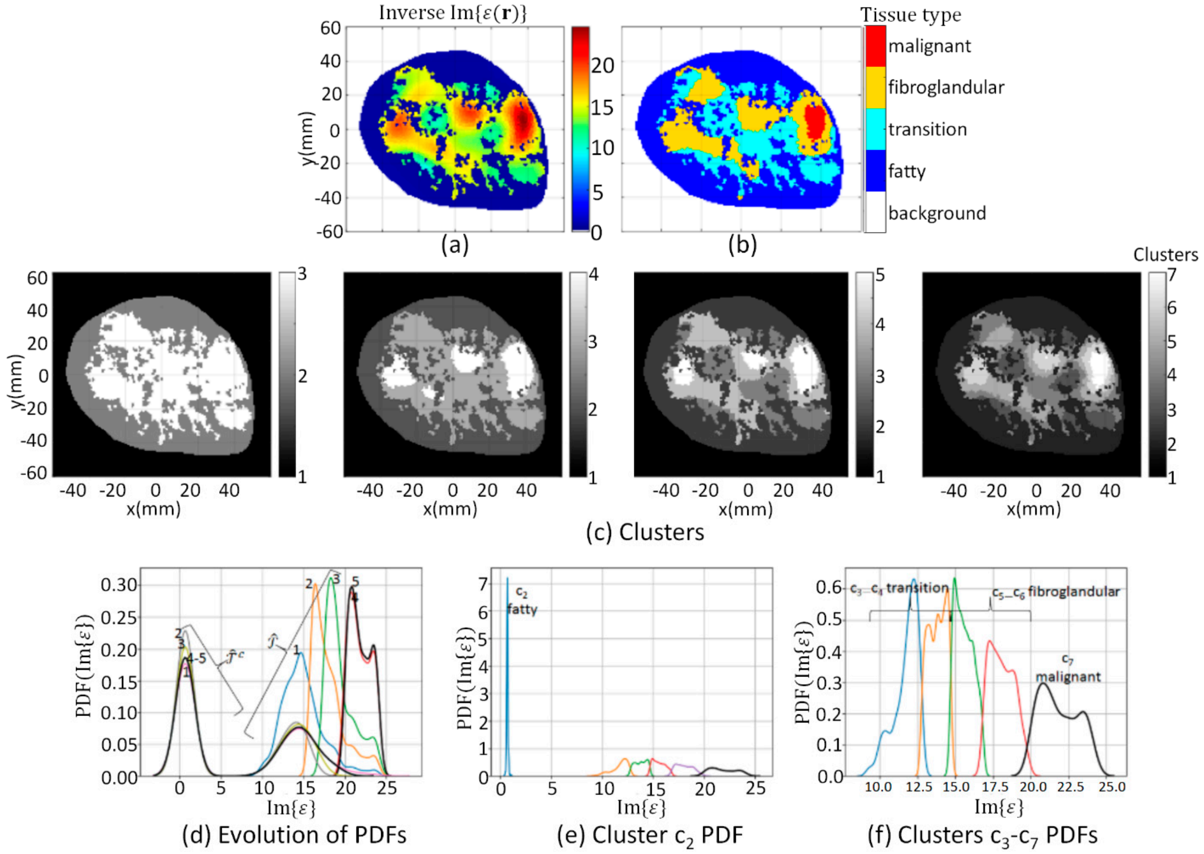

Figure S3. Case 3.1a Segmentation results of reconstruction derived from detailed internal structure prior- Imaginary component. (**a**) Region $\mathcal{R}$ extracted from imaginary component of inverse model; (**c**) Evolution of clusters at k = 3, 4, 5, and 7; (**d**) Evolution of PDF over data within $\hat{\mathcal{T}}^c$and $\hat{\mathcal{T}}$ where numbers indicate iteration; (**e**) PDF over data within cluster c_2, and (**f**) clusters c_3 (blue line) to c_7 (black line). Clusters $c_3 - c_4$ corresponds to transition tissue, $c_5 - c_6$ fibroglandular tissues, and c_7 corresponds to malignant tissue, which are mapped to segmentation masks leading to tissue type image (**b**).

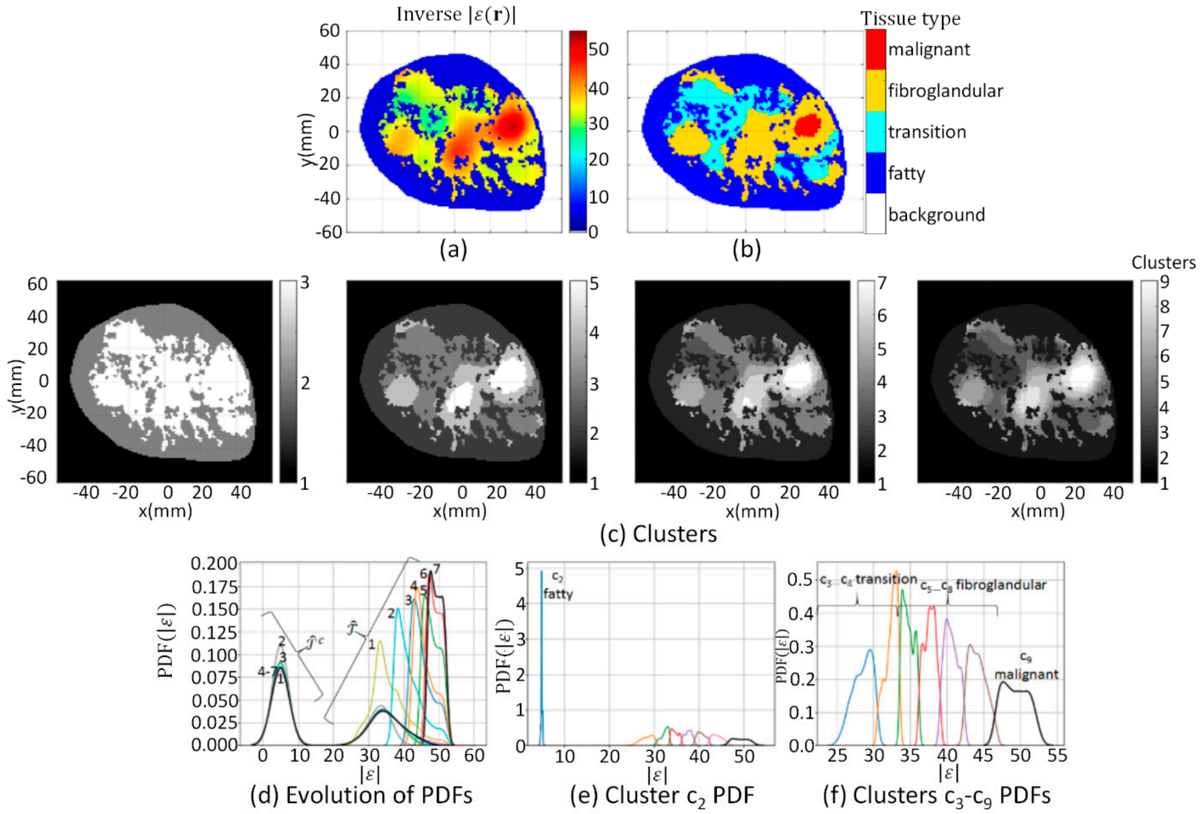

Figure S4. Case 3.1a Segmentation results of reconstruction derived from detailed internal structure prior-Magnitude. (**a**) Region $\mathcal{R}$ extracted from magnitude of inverse model complex permittivity; (**c**) Evolution of clusters at k = 3, 5, 7, and 9; (**d**) Evolution of PDF over data within $\hat{\mathcal{T}}^c$and $\hat{\mathcal{T}}$ where numbers indicate iteration; (**e**) PDF over data within cluster c_2, and (**f**) clusters c_3 (blue line) to c_9 (black line). Clusters $c_3 - c_4$ corresponds to transition tissue, $c_5 - c_8$ fibroglandular tissues, and c_9 corresponds to malignant tissue, which are mapped to segmentation masks leading to tissue type image (**b**).

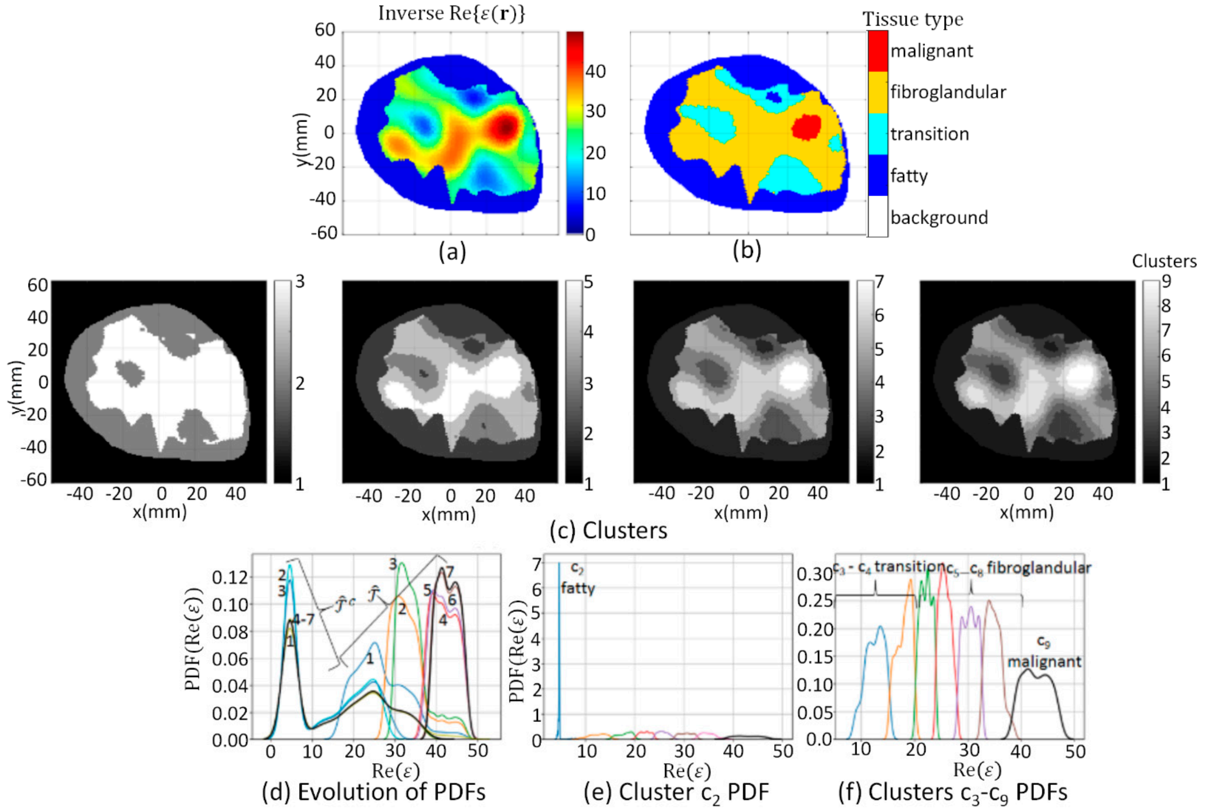

Figure S5. Case 3.1b Segmentation results of reconstruction derived from regional internal structure prior-Real component. (**a**) Region $\mathcal{R}$ extracted from real component of inverse model; (**c**) Evolution of clusters at $k = 3$, 5, 7, and 9; (**d**) Evolution of PDF over data within $\hat{\mathcal{T}}^c$ and $\hat{\mathcal{T}}$ where numbers indicate iteration; (**e**) PDF over data within cluster c_2, and (**f**) clusters c_3 (blue line) to c_9 (black line). Clusters $c_3 - c_4$ corresponds to transition tissue, $c_5 - c_8$ fibroglandular tissues, and c_9 corresponds to malignant tissue, which are mapped to segmentation masks leading to tissue type image (**b**).

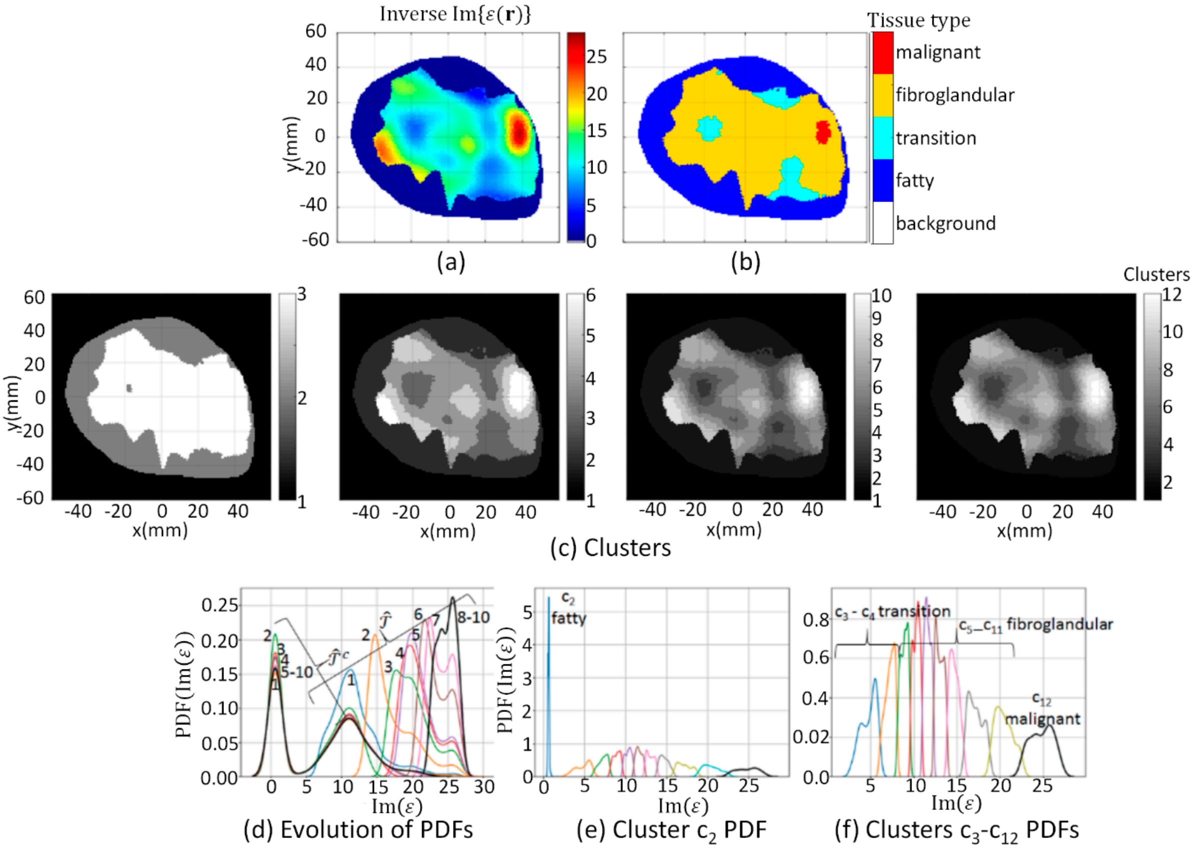

Figure S6. Case 3.1b Segmentation results of reconstruction derived from regional internal structure prior-Imaginary component. (**a**) Region $\mathcal{R}$ extracted from imaginary component of inverse model; (**c**) Evolution of clusters at $k = 3, 6, 10$, and 12; (**d**) Evolution of PDF over data within $\hat{\mathcal{T}}^c$and $\hat{\mathcal{T}}$ where numbers indicate iteration; (**e**) PDF over data within cluster c_2, and (**f**) clusters c_3 (blue line) to c_{12} (black line). Clusters $c_3 - c_4$ corresponds to transition tissue, $c_5 - c_{11}$ fibroglandular tissues, and c_{12} corresponds to malignant tissue, which are mapped to segmentation masks leading to tissue type image (**b**).

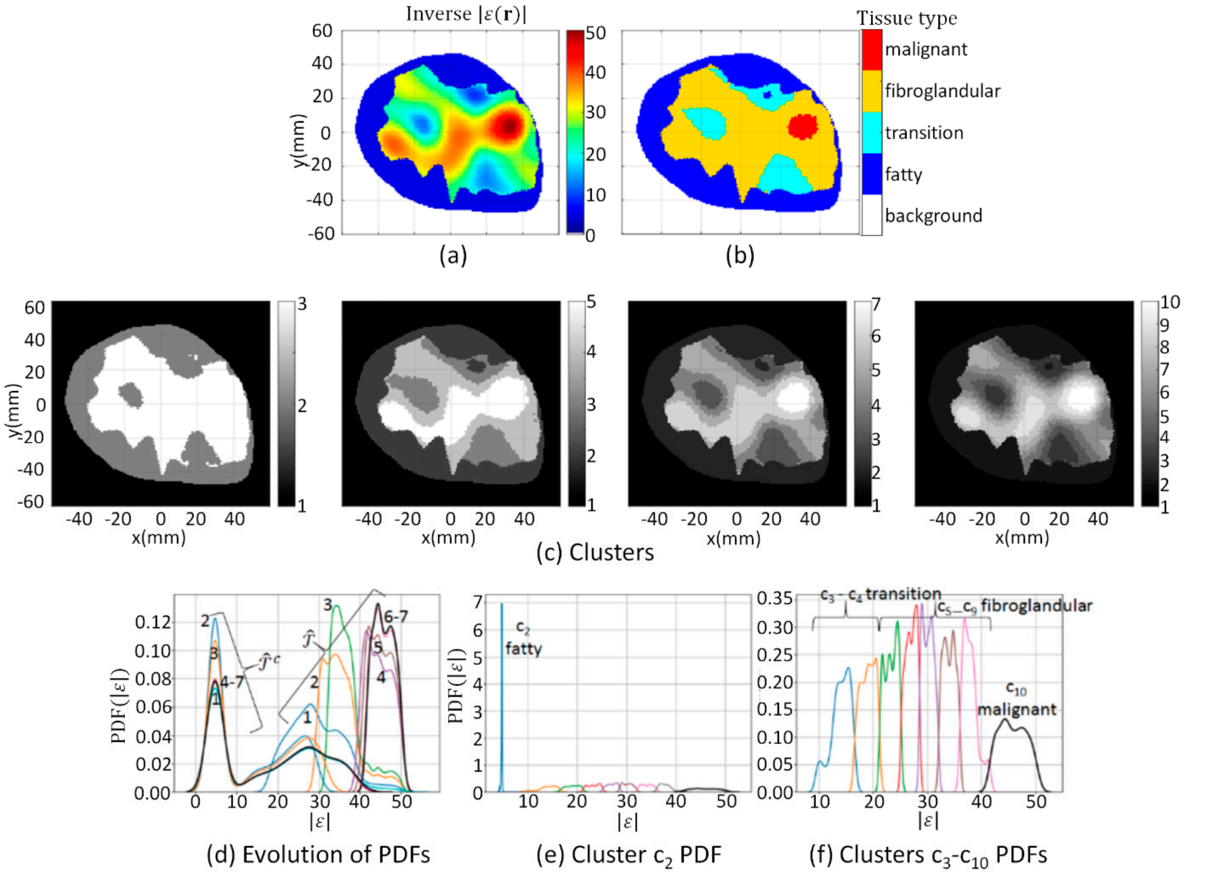

Figure S7. Case 3.1b Segmentation results of reconstruction derived from regional internal structure prior-Magnitude. (**a**) Region $\mathcal{R}$ extracted from magnitude of inverse model complex permittivity; (**c**) Evolution of clusters at k = 3, 5, 7, and 10; (**d**) Evolution of PDF over data within $\hat{\mathcal{T}}^c$and $\hat{\mathcal{T}}$ where numbers indicate iteration; (**e**) PDF over data within cluster c_2, and (**f**) clusters c_3 (blue line) to c_{10} (black line). Clusters $c_3 - c_4$ corresponds to transition tissue, $c_5 - c_9$ fibroglandular tissues, and c_{10} corresponds to malignant tissue, which are mapped to segmentation masks leading to tissue type image (**b**).

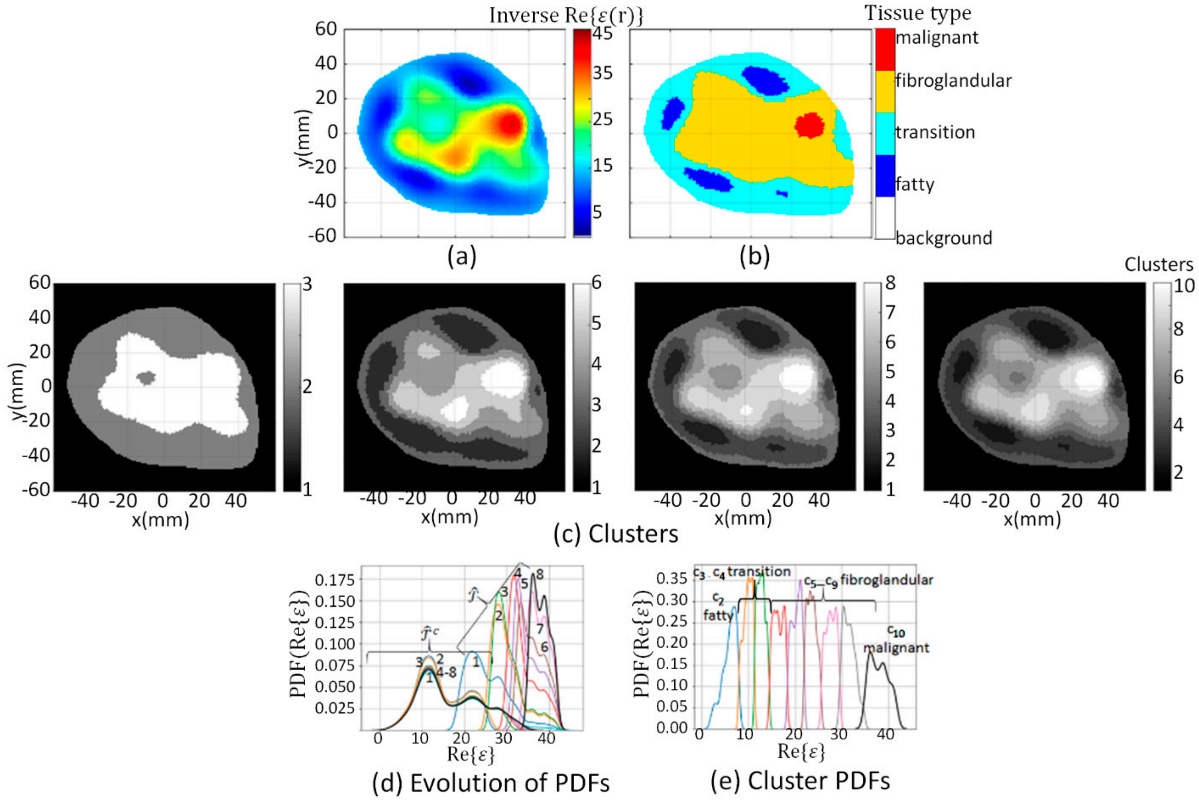

Figure S8. Case 3.1c Segmentation results of reconstruction derived from skin region prior-Real component. (**a**) Region $\mathcal{R}$ extracted from real component of inverse model; (**c**) Evolution of clusters at $k = 3$, 6, 8, and 10; (**d**) Evolution of PDF over data within $\hat{\mathcal{T}}^c$ and $\hat{\mathcal{T}}$ where numbers indicate iteration; (e) PDF over data within clusters c_2 (blue line) to c_{10} (black line). Cluster c_2 corresponds to fatty tissue, $c_3 - c_4$ transition tissue, $c_5 - c_9$ fibroglandular tissues, and c_{10} corresponds to malignant tissue, which are mapped to segmentation masks leading to tissue type image (**b**).

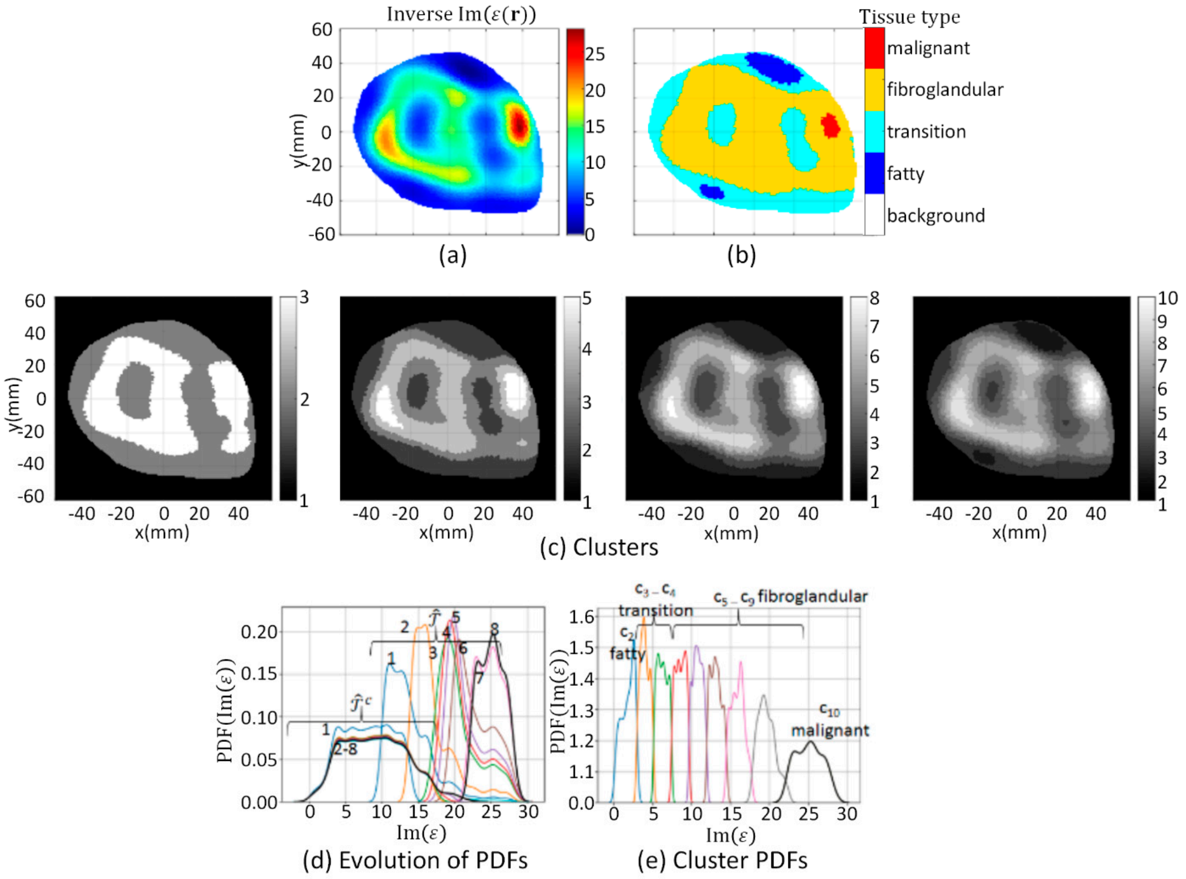

.

Figure S9. Case 3.1c Segmentation results of reconstruction derived from skin region prior-Imaginary component. (**a**) Region $\mathcal{R}$ extracted from imaginary component of inverse model; (**c**) Evolution of clusters at k = 3, 5, 8, and 10; (**d**) Evolution of PDF over data within $\hat{\mathcal{T}}^c$and $\hat{\mathcal{T}}$ where numbers indicate iteration; (e) PDF over data within clusters c_2 (blue line) to c_{10} (black line). Cluster c_2 corresponds to fatty tissue, $c_3 - c_4$ transition tissue, $c_5 - c_9$ fibroglandular tissues, and c_{10} corresponds to malignant tissue, which are mapped to segmentation masks leading to tissue type image (**b**).

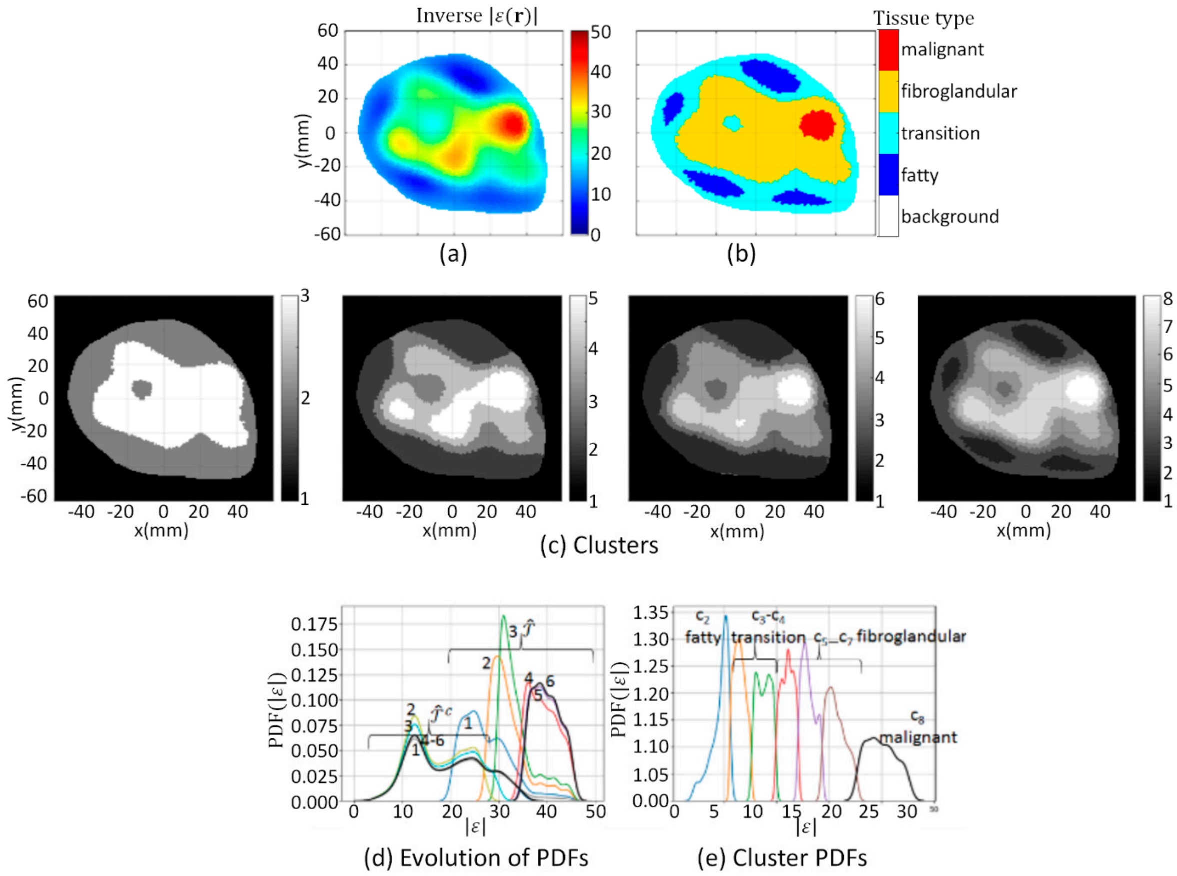

Figure S10. Case 3.1c Segmentation results of reconstruction derived from skin region prior-Magnitude. (**a**) Region $\mathcal{R}$ extracted from magnitude of inverse model complex permittivity; (**c**) Evolution of clusters at k = 3, 5, 6, and 8; (**d**) Evolution of PDF over data within $\hat{\mathcal{T}}^c$and $\hat{\mathcal{T}}$ where numbers indicate iteration; (e) PDF over data within clusters c_2 (blue line) to c_8 (black line). Cluster c_2 corresponds to fatty tissue, $c_3 - c_4$ transition tissue, $c_5 - c_7$ fibroglandular tissues, and c_8 corresponds to malignant tissue, which are mapped to segmentation masks leading to tissue type image (**b**).
